# Two neuroanatomical subtypes of males with autism spectrum disorder revealed using semi-supervised machine learning

**DOI:** 10.1186/s13229-022-00489-3

**Published:** 2022-02-23

**Authors:** Guanlu Liu, Liting Shi, Jianfeng Qiu, Weizhao Lu

**Affiliations:** 1 Department of Radiology, The Second Affiliated Hospital of Shandong First Medical University, Taian, China; 2 Department of Radiology, Shandong First Medical University and Shandong Academy of Medical Sciences, Taian, China; 3 Science and Technology Innovation Center, Shandong First Medical University and Shandong Academy of Medical Sciences, Jinan, China; 4 Department of Medical Imaging, Suzhou Institute of Biomedical Engineering and Technology, Chinese Academy of Science, Suzhou, China

**Keywords:** Autism, Magnetic resonance imaging, Semi-supervised machine learning, Neurosubtyping

## Abstract

**Background:**

Clinical and etiological varieties remain major obstacles to decompose heterogeneity in autism spectrum disorders (ASD). Recently, neuroimaging raised new hope to identify neurosubtypes of ASD for further understanding the biological mechanisms behind the disorder.

**Methods:**

In this study, brain structural MRI data and clinical measures of 221 male subjects with ASD and 257 healthy controls were selected from 7 independent sites from the Autism Brain Image Data Exchange database (ABIDE). Heterogeneity through discriminative analysis (HYDRA), a recently-proposed semi-supervised clustering method was utilized to divide individuals with ASD into several neurosubtypes by regional volumetric measures of gray matter, white matter, and cerebrospinal fluid. Voxel-wise volume, clinical measures, dynamic resting-state functional magnetic resonance imaging (R-fMRI) measures among different neurosubtypes of ASD were explored. In addition, support vector machine (SVM) model was applied to test whether the neurosubtyping of ASD could improve diagnostic accuracy of ASD.

**Results:**

Two neurosubtypes of ASD with different voxel-wise volumetric patterns were revealed. The full-scale intelligence quotient (IQ), verbal IQ, Autism Diagnostic Observation Schedule (ADOS) total scores and ADOS severity scores were significantly different between the two neurosubtypes, the total intracranial volume was correlated with performance IQ in Subtype 1 and was correlated with ADOS communication score and ADOS social score in Subtype 2. Compared with Subtype 2, Subtype 1 showed lower dynamic R-fMRI measures, lower dynamic functional architecture stability, higher mean and lower standard deviation (SD) of concordance among dynamic R-fMRI measures in cerebellum. In addition, classification accuracies between ASD neurosubtypes and healthy controls were significantly improved compared with classification accuracy between entire ASD group and healthy controls.

**Limitations:**

The present study excluded female subjects and left-handed subjects, which limited the ability to investigate the associations between these factors and the heterogeneity of ASD.

**Conclusions:**

The two distinct neuroanatomical subtypes of ASD validated by other data modalities not only adds reliability of the result, but also bridges from brain phenomenology to clinical behavior. The current neurosubtypes of ASD could facilitate understanding the neuropathology of this disorder and could be potentially used to improve clinical decision-making process and optimize treatment.

**Supplementary Information:**

The online version contains supplementary material available at 10.1186/s13229-022-00489-3.

## Background

Autism spectrum disorder (ASD), as an increasingly common neurodevelopmental disorder, affects 1–2% of the general population [1, 2]. ASD is characterized by impairments in social cognition as well as restricted and repetitive behaviors (RRB) [3]. However, different from other psychiatric disorders characterized by symptom severity, patients with ASD display a broad range of behavior types and severities [4]. For instance, verbal and nonverbal intelligence quotient (IQ) are highly variable in patients with ASD [5] and RRB can range from low-level stereotyped motor behaviors to higher order behaviors such as insistence on sameness in ASD patients [3]. ASD patients are frequently associated with comorbid disorders such as language skills or coordination disorders, or attention deficit and hyperactivity disorder [6]. Besides, more than 100 genes [7] and many aspects of brain structure have been associated with ASD [8]. The high biological and clinical heterogeneity of ASD patients have hindered attempts at understanding the neurobiological mechanisms of the disorder [9]. Until recently, the analysis of ASD mainly depends on the spectrum of symptom severity [10], while the results of these efforts have been neither distinguishable nor fully reflecting the underlying biology [11]. Thus, the DSM-5 have replaced subcategories of autism into a single diagnostic category of ASD [3]. Despite the cancellation of ASD subtypes, subtyping of ASD has several clinical benefits, such as early and accurate detection, developmental trajectories, and response to treatment.

Notably, the development of ASD has been associated with abundant heterogeneity and this developmental heterogeneity is manifested in head circumference and total brain volume [12–14]. However, abnormal increase of head circumference cannot be observed in all subjects with ASD, one research implies that about 80% of subjects with ASD have no clinically enlarged brain [15]. Hence, the abnormal development of head circumference in patients with ASD might represent a potential neurological subtype of ASD [16]. Recently, neuroimaging offered new possibilities to further understand the biological mechanisms behind ASD. A recent cluster analysis based on whole-brain voxel-based morphometry (VBM) obtained 3 ASD neurosubtypes [17]. However, different numbers of neurosubtypes have been reported by previous studies based on the neuroimaging features including cortical thickness and regional volumes [18], or intrinsic functional connectivity based on cortical region of interests (ROIs) [19]. The diverse results of these studies may be mainly due to the use of different image features. In addition, the differences in these findings may be due to factors such as sample size, sex ratio and clustering algorithm, etc.

In order to overcome the high heterogeneity of ASD, several unsupervised clustering methods have been used [20]. Different from the unsupervised clustering methods in previous studies [20], heterogeneity through discriminative analysis (HYDRA) is one of the first algorithms to explore anatomical heterogeneity by supervised clustering with adjustable number of clusters. HYDRA not only inherits the ability of non-linear kernel classification methods to accurately fit to heterogeneous data in terms of disease prediction, but also provides explicit clustering information that can be used to determine subtypes of pathology [21]. To date, HYDRA has been successfully used in the subtype of Alzheimer's disease [21] and schizophrenia [22]. We hypothesized that this advanced clustering method would effectively reveal distinct neurosubtypes of ASD and build stable brain-behavior relationships which could potentially be used to improve clinical decision-making process and optimize treatment in the future. Therefore, in this study, HYDRA was used to classify male ASD patients into distinct neurosubtypes with regional volumetric measures of gray matter, white matter, and cerebrospinal fluid (CSF) from structural magnetic resonance imaging (MRI), brain-behavior relationships were assessed in different neurosubtypes. In addition, dynamic resting-state functional magnetic resonance imaging (R-fMRI) and machine learning-based classification were used to test the rationality of the neurosubtypes.

## Materials and methods

### Datasets and participant selection

Publicly-available MRI data and phenotype data were downloaded from the Autism Brain Image Data Exchange database (ABIDE) (http://fcon_1000.projects.nitrc.org/indi/abide/). In order to improve the reliability and repeatability of the results, a strict data exclusion scheme was adopted in this study. Data curation was conducted using the following exclusion criteria: (1) female subjects; (2) left or mixed handedness or subjects with no handedness information; (3) subjects with no full-scale IQ information or full-scale IQ below 80; (4) to reduce ageing effects, subjects older than 45 years of age were excluded; (5) an initial quality check was performed by an experienced radiologist, then the Image Quality Rate (IQR) in CAT12 software (Computational Anatomy Toolbox 12, CAT12, http://www.neuro.uni-jena.de/cat/) was used in the quality control of structural images to avoid the subjectivity of manual check. As low image quality can lead to underestimation of the gray matter in most preprocessings [23], therefore, we adopted strict image quality control procedures, and subjects with IQR lower than B- were excluded (The detailed image quality rating criterion and quality control steps are shown in Additional file 1: Fig. S1 and Supplementary Text. In addition, Additional file 1: Table S1 demonstrates the quality control metrics for the enrolled subjects, no significant differences were found in terms of image quality between health controls and ASD patients). (6) Study sites with less than 20 ASD patients were excluded. In addition, the New York University Medical Center (NYU) sample 2 in ABIDE-II databases were excluded because different scanning parameters compared with NYU sample in ABIDE-I and NYU sample 1 in ABIDE-II. The individuals scanned using a head coil with 32 channels in Kennedy Krieger Institute (KKI) sample of ABIDE-II were excluded because the rest KKI data from ABIDE-I and ABIDE-II were scanned using a head coil with 8 channels. After applying these exclusion criteria, data of 478 subjects from ABIDE-I and ABIDE-II from 7 sites remained. Demographics for the resultant sample (221 ASD patients and 257 healthy controls) are presented in Table 1.

**Table 1:** Demographic and clinical data

	Site 1 (GU)		Site 2 (KKI)		Site 3 (OHSU)		Site 4 (PITT)		Site 5 (TRINITY)		Site 6 (UCLA)		Site 7 (USM)	
	HC	ASD	HC	ASD	HC	ASD	HC	ASD	HC	ASD	HC	ASD	HC	ASD
*N*	22	25	66	28	41	35	21	20	25	22	35	38	44	53
Type (A/AS/P/NA)	**–**	0/0/0/25	**–**	11/16/1/0	**–**	0/0/0/35	**–**	20/0/0/0	**–**	8/7/7/0	**–**	32/0/0/6	**–**	45/0/1/7
Age	10.8 ± 1.7	11.1 ± 1.7	10.5 ± 1.3	10.3 ± 1.6	10.3 ± 1.5	11.7 ± 2.1	19.3 ± 7.1	19.9 ± 7.2	17.1 ± 3.8	17.1 ± 3.6	12.8 ± 2.3	13.0 ± 2.3	21.6 ± 7.5	22.1 ± 7.1
IQ														
FIQ	121 ± 12.0	121 ± 12.9	114 ± 11.2	106 ± 14.1	114 ± 13.1	109 ± 15.5	110 ± 9.1	113 ± 14.1	111 ± 12.2	110 ± 13.1	107 ± 11.2	103 ± 11.4	115 ± 13.3	104 ± 15.1
VIQ	122 ± 13.0	123 ± 11.5	119 ± 11.0	113 ± 15.0	**–**	**–**	108 ± 10.3	110 ± 13.0	110 ± 13.7	109 ± 14.0	109 ± 11.3	105 ± 13.2	113 ± 13.5	100 ± 16.1
PIQ	117 ± 15.0	117 ± 14.2	113 ± 12.9	107 ± 15.0	**–**	**–**	108 ± 10.0	112 ± 14.3	110 ± 9.1	110 ± 10.9	104 ± 12.3	101 ± 13.2	114 ± 13.3	107 ± 14.9
ADOS														
Total	**–**	10.3 ± 5.0	**–**	10.6 ± 1.8	**–**	9.0 ± 3.3	**–**	12.0 ± 3.1	**–**	10.3 ± 2.9	**–**	11.1 ± 3.7	**–**	12.5 ± 3.1
Comm	**–**	2.9 ± 1.7	**–**	3.2 ± 0.6	**–**	2.8 ± 1.3	**–**	4.1 ± 1.0	**–**	**–**	**–**	3.3 ± 1.5	**–**	4.3 ± 1.4
Social	**–**	7.4 ± 3.8	**–**	7.4 ± 1.5	**–**	6.2 ± 2.2	**–**	8.0 ± 2.4	**–**	**–**	**–**	7.8 ± 2.7	**–**	8.2 ± 2.3
RRB	**–**	1.6 ± 1.6	**–**	2.8 ± 1.6	**–**	2.1 ± 1.1	**–**	2.5 ± 1.5	**–**	**–**	**–**	1.7 ± 1.8	**–**	1.6 ± 1.5
Severity	**–**	5.7 ± 2.7	**–**	6.9 ± 2.0	**–**	7.2 ± 1.3	**–**	**–**	**–**	**–**	**–**	6.9 ± 2.3	**–**	8.1 ± 2.0
ADI-R														
Social	**–**	19.0 ± 4.9	**–**	19.8 ± 6.1	**–**	20.1 ± 5.1	**–**	20.9 ± 3.0	**–**	20.2 ± 6.3	**–**	20.4 ± 4.7	**–**	**–**
Verbal	**–**	14.3 ± 3.9	**–**	15.4 ± 4.7	**–**	16.8 ± 4.4	**–**	15.8 ± 3.7	**–**	16.4 ± 5.0	**–**	16.4 ± 4.6	**–**	**–**
RRB	**–**	5.0 ± 2.3	**–**	6.4 ± 2.3	**–**	6.3 ± 2.6	**–**	6.6 ± 2.4	**–**	5.6 ± 2.7	**–**	7.2 ± 2.2	**–**	**–**
SRS														
Total	20.1 ± 16.0	86.2 ± 35.3	16.4 ± 9.5	93.6 ± 32.3	20.2 ± 15.7	95.3 ± 27.1	**–**	**–**	**–**	**–**	**–**	**–**	16.8 ± 13.1	95.8 ± 33.6
Aware	5.0 ± 3.3	10.8 ± 3.8	4.0 ± 2.4	13.8 ± 3.7	4.0 ± 2.7	13.1 ± 3.6	**–**	**–**	**–**	**–**	**–**	**–**	4.6 ± 2.9	12.4 ± 3.2
Cognit	2.6 ± 2.2	16.2 ± 5.1	2.5 ± 2.3	16.2 ± 7.2	3.5 ± 3.8	14.7 ± 6.0	**–**	**–**	**–**	**–**	**–**	**–**	3.0 ± 2.0	17.4 ± 6.5
Comm	6.6 ± 6.8	28.4 ± 11.0	4.8 ± 3.8	32.1 ± 10.6	6.4 ± 5.5	32.8 ± 11.0	**–**	**–**	**–**	**–**	**–**	**–**	7.0 ± 5.1	37.0 ± 12.9
Motiva	3.9 ± 3.3	13.0 ± 5.6	3.4 ± 2.6	13.6 ± 7.9	3.9 ± 3.3	14.8 ± 5.8	**–**	**–**	**–**	**–**	**–**	**–**	4.6 ± 5.1	19.1 ± 8.1
Manner	2.0 ± 3.4	15.4 ± 6.0	1.7 ± 1.7	17.9 ± 7.5	2.3 ± 3.1	20.0 ± 5.7	**–**	**–**	**–**	**–**	**–**	**–**	2.4 ± 2.9	22.4 ± 6.9

Abbreviations: GU, Georgetown University; KKI, Kennedy Krieger Institute; OHSU, Oregon Health and Science University; PITT, University of Pittsburgh School of Medicine; TRINITY, Trinity Centre for Health Sciences; UCLA, University of California, Los Angeles; USM, University of Utah School of Medicine; HC, healthy control; ASD, autism spectrum disorder; N, number; A, autism type; AS, Asperger syndrome type; P, pervasive developmental disorder type; NA, not applicable; ADOS, autism diagnostic observation schedule; IQ, intelligence quotient; FIQ, full-scale IQ; VIQ, verbal IQ; PIQ, performance IQ; Comm, communication, RRB, stereotyped behaviors and restricted interests; ADOS Total = social + communication; ADI-R, autism diagnostic interview revised; SRS, social responsiveness scale; Aware, awareness; Cognit, cognition; Motiva, motivation; Manner, mannerisms; SRS Total = awareness + cognition + communication + motivation + mannerisms

### Image preprocessing

At first, CAT12 was applied for VBM preprocessing to structural MRI data from each subject. The main steps applied to the structural MRI data were as follows: (1) normalization of T1 image into the Montreal Neurological Institute (MNI) space and the voxel size was resampled into 1.5 × 1.5 × 1.5 mm^3^; (2) segmentation of the normalized images into gray matter, white matter and CSF; (3) modulation to convert the voxel values of tissue concentration (density) to volume; (4) calculation of the volume value based on the ROI from gray matter, white matter and CSF; (5) smoothing with an 8-mm full width at half maximum (FWHM) isotropic Gaussian kernel.

### Subtyping ASD with HYDRA

For each subject, we obtained volumes of 142 ROIs (The ROIs were derived from the Neuromorphometrics atlas, http://www.neuromorphometrics.com/, detailed information of the ROIs is listed in Additional file 1: Table S3) from structural MRI data as features for further cluster analysis. Prior to subtyping, manual check was carried out to check the alignment between the atlas and the normalized volumetric maps, and to eliminate any absurd values of volumes (Details are shown in the Supplementary Text). Then the effect of age and site-specific factors on the ROI volumes were estimated using a linear model and were regressed out [17]. The covariate regression strategy was also applied to further voxel-wise volumetric and dynamic R-fMRI measures analysis of ASD subtypes, as well as classification between ASD subtypes and healthy controls.

HYDRA was utilized based on the volumetric measures of the ROIs to identify ASD subtypes [21]. HYDRA consists of the following steps: Firstly, ASD subjects are given negative labels and healthy controls are given positive labels. HYDRA will determine the number of hyperplanes by the K value of the clusters number to generate convex polyhedron for separating ASD patients from the healthy controls. An extending standard linear maximum margin classifiers is introduced to calculate the distance from each ASD subject to each hyperplane, and ASD subjects will be assigned to the hyperplane closest to themselves, so that all ASD subjects are divided into K clusters. Following parameters were used to ensure convergent and stable clustering results, and to alleviate computational burden: 50 iterations between estimating hyperplanes and cluster estimation, 20 clustering consensus steps, regularization parameter of 0.25, 10 cross-validation folds and clustering range from 2 to 8.

In this experiment, the above process runs within the framework of tenfold cross-validation. For each time, 9 folds subjects are selected for the above clustering process. The process of 50 times iterations is adopted to find the optimal convex plane for clustering estimation. Finally, for each ASD subject, it participated in 9 clustering processes and obtained 9 same or different clustering labels. The 20 clustering consensus steps will determine the final cluster label of each ASD by a cooccurrence matrix generated from the labels of 9 clustering processes. Meanwhile, the algorithm quantifies the similarity between clustering results in a 10-folds cross-validated fashion by the adjusted rand index (ARI) [24] to assess the clustering stability. ARI evaluates the contingency of grouping and provides a more conservative overlap estimation. The values of ARI range from 0 to 1, and the ARI value of 1 represents a perfect clustering. A schematic illustration of the HYDRA method is shown in Additional file 1: Fig. S3.

### Reproducibility analysis of ASD subtypes

To assess the reproducibility of subtypes, we took a series of analyses, including split-sample tests [25, 26] and leave-one-site-out validation [27]. In order to evaluate the reproducibility of ASD subtypes, we conducted a split-sample test analysis. The healthy controls and patients were randomly divided into two parts and then HYDRA was applied in these two parts, respectively. Age was matched between healthy controls and ASD in the two splits (Additional file 1: Table S4). Voxel-wise volumetric maps were further compared between healthy controls and each ASD subtype in the two splits.

In addition, the subtypes were further validated using a leave-one-site-out strategy. In this strategy, the data of 6 sites were used to train HYDRA models and subtype labels of the last remaining sites were identified by the trained models. This procedure was repeated 7 times to complete possible combinations for all sites. In other words, in each leave-one-site-out process, one site was regarded as an independent site, and the predicted labels of this site was determined by the trained model from the other 6 sites. Finally, the predicted labels of leave-one-site-out strategy from all 7 sites were compared with the original labels obtained by taking all 7 sites together. The voxel-wise gray matter volumetric maps were estimated using leave-one-site-out-predicted results between each subtype and healthy controls.

### Voxel-wise volumetric analysis of ASD subtypes

We conducted voxel-wise volumetric analyses using regionally linear multivariate discriminative statistical mapping (MIDAS) [28] to explore the alterations of gray and white volume in ASD subtypes with age and site as covariates. Compared with other information-mapping methods, MIDAS effectively determines the regionally varying, anisotropic filtering of any image data that optimally captures group differences [28]. Voxel-wise group (ASD subtypes and healthy control) comparisons of the smoothed gray and white matter volume maps were performed using two sample t-tests within SPM12. Similarly, in this step age and site were included in a general linear model as covariates. Then, the t-statistic maps were converted to the effect size (Cohen’s d) maps. Finally, the voxel-wise statistical significance values (p-values) were corrected by false discovery rate (FDR) (FDR-p < 0.05) and were then used as a mask to show the effect size maps between the groups via MIDAS.

### Clinical examination of ASD subtypes

First, we verified that subtypes were composed of similar proportions of ASD patients per site based on the chi-square test. To assess whether severity of ASD differed between subtypes, a two-sample t-test was utilized on the full-scale IQ, verbal IQ, performance IQ, Autism Diagnostic Observation Schedule (ADOS) total, social, communication, RRB, severity scores, Autism Diagnostic Interview Revised (ADI-R) Social, Verbal, RRB, Social Responsiveness Scale (SRS) total, awareness, cognition, communication, motivation, and mannerisms respectively. Given a widely hypothesized characteristic on verbal and non-verbal IQ discrepancy in ASD [29, 30], the verbal IQ and performance IQ was included. Besides, within each subtype, the relationship between total intracranial volume and above demographic and clinical data was assessed using Pearson’s correlation. In this procedure, subjects with missing ADOS measures were excluded.

### Dynamic R-fMRI measures analysis of ASD subtypes

In order to verify whether the two neuroanatomical subtypes of ASD were differed in brain functions, we analyzed R-fMRI data from the same cohorts included in the structural MRI dataset. In this step, subjects with head movements greater 2.0 mm of translation or 2.0 degrees of rotation in any direction were excluded. All dynamic R-fMRI measures were calculated via Data Processing & Analysis for Brain Imaging (DPABI, http://rfmri.org/DPABI). At first, we obtained 4 dynamic R-fMRI measures including regional homogeneity (ReHo), voxel-mirrored homotopic connectivity (VMHC), network degree centrality (DC) and global signal correlation (GSCorr). To characterize the dynamic R-fMRI measures, we computed the standard deviation (SD) map across time windows of each measure for subsequent statistical analysis. Besides, we calculated Kendall’s coefficient of concordance (KCC) for the 4 dynamic R-fMRI measures across time windows as the dynamic volume-wise concordance. The mean and SD of the time series of subjects’ dynamic volume-wise concordance index (one for each subject) were performed using two-sample t-tests, age, site, and head motion (mean framewise-displacement, FD) were treated as covariates by the general linear model. Finally, we calculated the voxel-to-atlas KCC mapping, which characterizes the stability of dynamic functional architecture. The detailed preprocessing and dynamic R-fMRI measures calculation steps are shown in Supplementary Text. Voxel-wise group comparisons (between Subtype 1 and Subtype 2) of the above-mentioned R-fMRI measures mapping were performed using two sample t-tests within SPM 12, age, site, and head motion (mean FD) were included in the general linear model as covariates. Gaussian Random Field (GRF) correction was performed to control false positives (voxel-level *p* < 0.001, to cluster-level *p* < 0.01, two-tailed). In addition, we also performed comparisons in dynamic R-fMRI measures between healthy controls and ASD subtypes.

### Classification between subtypes of ASD and healthy controls

To test whether subtyping could improve diagnostic label accuracy beyond group average comparisons, and to identify possible biomarkers from brain regions with volumetric differences, the support vector machine (SVM) model was applied to classify ASD and healthy controls based on the volumetric measures of the ROIs. Classification was performed between different subtypes of ASD patients and healthy controls respectively. In addition, classification was also performed between all ASD patients and healthy controls to test the effect of subtyping on improving classification accuracy between each subtype and the healthy control group. Notably, we ignored the feature selection step and chose a linear kernel to obtain feature weighting values. Since the weighting values indirectly represent the importance of each feature in the classification process, we applied weighting values of each feature to further explore meaningful ROIs in subtypes, respectively.

Since there were more healthy controls than ASD and subtypes of ASD, which would lead to the problem of unbalanced classification. We randomly matched the numbers of healthy controls and ASD subtypes and the process of number matching was repeated 10 times to eliminate random bias. Finally, we performed tenfold cross-validation in each number matching and the hyperparameters were selected in train setting for each fold by a tenfold cross-validation. The final accuracy was defined as the average accuracy of the 10-time matching.

## Results

### Two subtypes of ASD based on structural MRI

In this study, we evaluated the consistency of clustering assignment by adjusting the number of clusters from 2 to 8 using ARI. The maximum ARI value was found at *K* = 2 (ARI = 0.82), which indicated that the ASD samples were optimally partitioned by 2 subtypes based on the volumes of anatomical ROIs. The clustering results of HYDRA are shown in Additional file 1: Fig. S4. There were 115 ASD patients assigned into Subtype 1 and 106 ASD patients assigned into Subtype 2.

### Altered volumes in two ASD subtypes

**Fig. 1:**
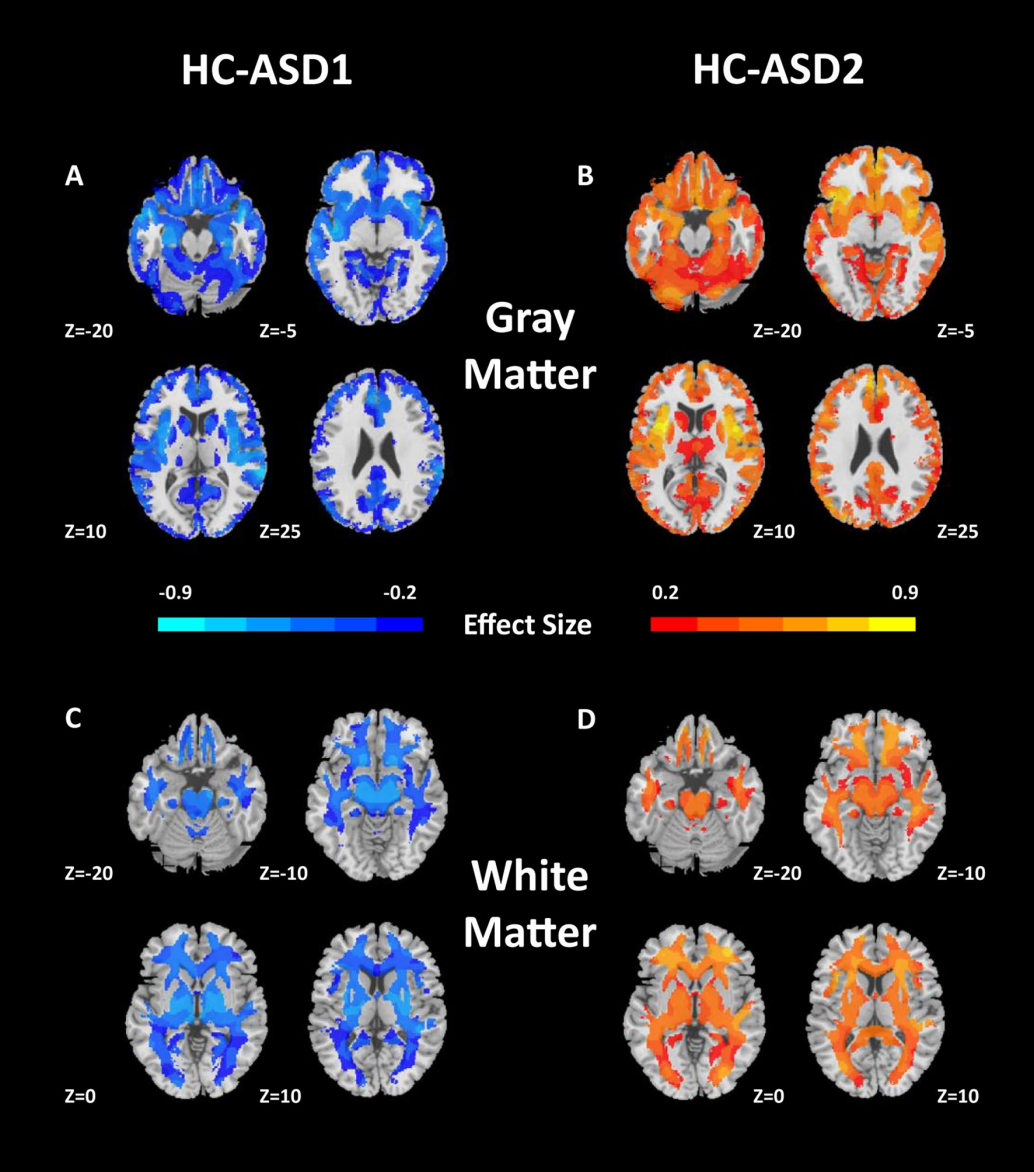
Patterns of gray and white matter volumes in the two ASD subtypes. Compared with healthy controls (HC), **A** ASD1 exhibits widespread patterns of increased gray matter volumes, **B** ASD2 exhibits widespread patterns of reduced gray matter volumes, **C** ASD1 shows increased white matter volumes, **D** ASD 2 shows reduced white matter volumes. Effect size (Cohen’s d) maps were generated from regional volumetric maps masked by the set of regions that showed statistically significant differences (P_FDR_ < 0.05) in the MIDAS analysis

Compared with healthy controls, ASD patients showed both increased and decreased gray matter and white matter volume by standard case–control test (Additional file 1: Fig. S5). HYDRA effectively subdivides the above effects and further revealed the possible neuroanatomical subtypes behind the complex brain volume changes. Subtypes showed marked differences in their voxel-wise patterns of neuroanatomical deficits. Subtype 1 showed an extensive gray and white matter volume increase compared with healthy controls (Fig. 1). In contrast, Subtype 2 showed an extensive gray and white matter volume decrease compared with healthy controls (Fig. 1).

### Clinical examination across two subtypes

**Fig. 2:**
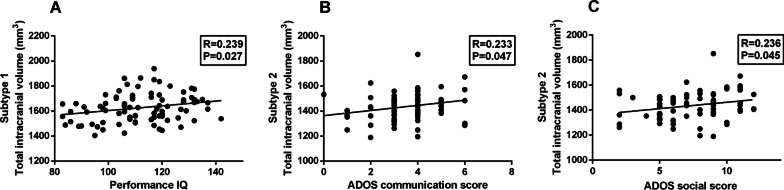
Associations between total intracranial volume and clinical measures in the two subtypes. **A** Total intracranial volume is correlated with performance IQ in Subtype 1 (*r* = 0.239 and *p* = 0.027); **B** Total intracranial volume is correlated with ADOS communication score in Subtype 2 (*r* = 0.233 and *p* = 0.047); **C** Total intracranial volume is correlated with ADOS social score in Subtype 2 (*r* = 0.236 and *p* = 0.045)

There are no significant differences in the site composition of two subtypes based on chi-square test (*χ*^2^ = 3.141, *df* = 6, *p* = 0.791). Using two-sample *t* test, we discovered that full-scale IQ (*p* < 0.001, *T* = 3.672), performance IQ (*p* < 0.001, *T* = 3.90), ADOS total score (*p* = 0.041, *T* = 2.064) and ADOS severity scores (*p* = 0.004, *T* = 2.959) were lower in Subtype 2, but the two subtypes did not differ in age, verbal IQ, communication score and RRB score (Table 2 and Additional file 1: Table S5). The total intracranial volume was positively correlated with performance IQ in Subtype 1. Besides, the total intracranial volume was positively correlated with ADOS communication score (*r* = 0.233; *p* = 0.047) and ADOS social score (*r* = 0.236, *p* = 0.045) in Subtype 2 (Fig. 2).

**Table 2:** Demographic and clinical profiles across subgroups

	Subtype 1 (*n* = 115)	Subtype 2 (*n* = 106)	HC (*n* = 257)	*p* value ^a^	*p* value ^b^	*p* value ^c^
Age	15.3 ± 6.8	15.5 ± 6.0	14.2 ± 6.1	0.825	0.104	0.056
Type (A/AS/P/NA)	58/11/5/41	58/12/4/32	–	0.926	**–**	**–**
IQ						
FIQ	111 ± 15.3	104 ± 13.5	114 ± 12.1	< 0.001	0.182	< 0.001
VIQ	109 ± 15.7	107 ± 16.1	114 ± 13.0	0.545	0.006	0.001
PIQ	112 ± 14.3	103 ± 13.7	111 ± 13.0	< 0.001	0.714	< 0.001
Site	14/17/21/10/10/18/25	11/11/14/10/12/20/28	22/66/41/21/25/35/47	0.791	–	–
ADOS						
Total	11.8 ± 3.6	10.7 ± 3.3	1.3 ± 1.7	0.041	< 0.001	< 0.001
Comm	3.7 ± 1.6	3.5 ± 1.3	0.6 ± 1.0	0.469	< 0.001	< 0.001
Social	8.1 ± 2.5	7.4 ± 2.6	0.7 ± 1.0	0.111	< 0.001	< 0.001
RRB	2.1 ± 1.8	1.7 ± 1.5	0.1 ± 0.5	0.090	< 0.001	< 0.001
Severity	7.7 ± 1.5	6.4 ± 2.4	**–**	0.004	**–**	**–**
ADI-R						
Social	20.5 ± 5.0	19.5 ± 5.2	**–**	0.209	–	–
Verbal	16.3 ± 4.3	15.4 ± 4.6	**–**	0.219	–	–
RRB	6.1 ± 2.5	6.4 ± 2.5	**–**	0.517	–	–
SRS						
Total	96.1 ± 32.8	90.5 ± 32.0	17.7 ± 12.9	0.342	< 0.001	< 0.001
Aware	13.2 ± 4.0	11.7 ± 3.4	4.3 ± 2.7	0.096	< 0.001	< 0.001
Cognit	16.6 ± 6.2	14.9 ± 5.8	2.8 ± 2.7	0.223	< 0.001	< 0.001
Comm	33.8 ± 10.1	28.9 ± 11.8	5.7 ± 5.1	0.058	< 0.001	< 0.001
Motiva	15.5 ± 5.8	12.9 ± 7.3	3.7 ± 3.1	0.095	< 0.001	< 0.001
Manner	18.9 ± 6.3	17.3 ± 7.1	1.9 ± 2.5	0.316	< 0.001	< 0.001

The *p* values were calculated using a two-sample *t* test except for the type and site composition (^a^Subtype1 vs. Subtype2; ^b^HC vs. Subtype1; ^c^HC vs. Subtype2). Abbreviations: IQ, intelligence quotient; FIQ, full-scale IQ; VIQ, verbal IQ; PIQ, performance IQ; ADOS, autism diagnostic observation schedule; Social, social interaction; Comm, communication. RRB, restricted repetitive behavior. ADOS Total = social interaction + communication; ADI-R, autism diagnostic interview revised; SRS, Social Responsiveness Scale; Aware, awareness; Cognit, cognition; Motiva, motivation; Manner, mannerisms; The number of ASD patients with IQ, ADOS scores, ADI-R scores, and SRS scores available are described in the Supplement Table S2

### The results of reproducibility analysis

**Fig. 3:**
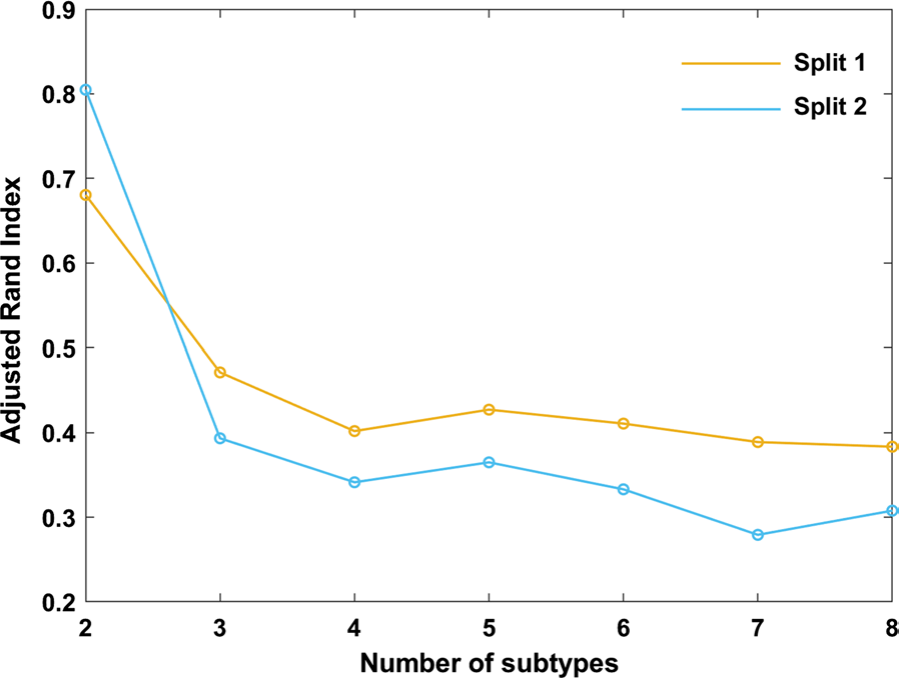
Cross-validated stability of split-half samples. Results indicate that *K* = 2 yields highly reproducible subtypes in both Split 1 and Split 2

**Fig. 4:**
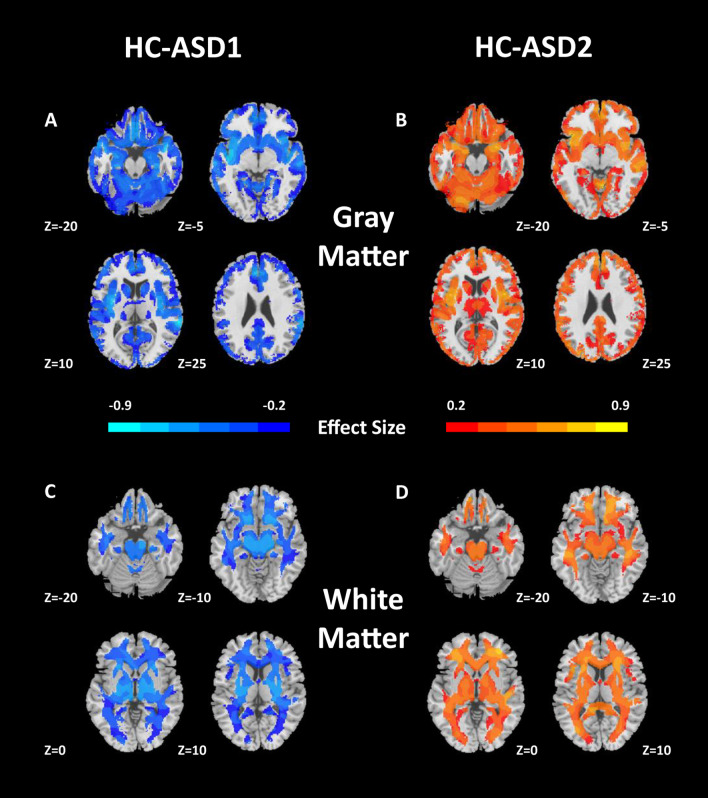
GM and WM volumetric differences between each subtype and healthy controls (HC) in leave-one-site-out analysis (*K* = 2, *P*_FDR_ < 0.05). These results are consistent with those obtained using the entire sample together. Abbreviations: GM, gray matter; WM, white matter

The superior reproducibility of ASD subtypes were shown in the split-sample test. (Fig. 3). The voxel-wise volumetric patterns were also reproducible between the two halves in the split-half test at 2 subtypes (Additional file 1: Fig. S6 and Additional file 1: Fig. S7). Furthermore, the reproducibility analyses of the subtypes were carried out using the leave-one-site-out cross-validation. When the number of clusters was set to 2, the predicted labels of 2 subtypes from all 7 sites using leave-one-site-out were compared with the original assignments obtained by taking all the sites together. The percentage overlap of patients that were assigned to the same subtype was 87.78% (92% in Site 1, 82.14% in Site 2, 97.14% in Site 3, 75% in Site 4, 95.46% in Site 5, 86.84% in Site 6 and 84.91% in Site 7, Additional file 1: Fig. S8). The analysis of voxel-wise gray and white matter volumetric maps was consistent with the original experiment (Fig. 4).

### Dynamic R-fMRI differences

After exclusion, 87 Subtype 1 and 81 Subtype 2 of ASD were remained. By two-sample t-test between ASD subtypes, we discovered that Subtype 1 showed lower dynamic R-fMRI measures including ReHo, DC and GSCorr mainly in the cerebellum (Additional file 1: Fig. S9). We tested the difference between ASD subtypes for mean and SD of concordance time series. The results of two sample t-test are shown in the Additional file 1: Fig. S10. The mean of concordance index of ASD subtype 1 is significantly higher than Subtype 2 (*p* < 0.001, *T* = 4.071), but SD is significantly lower than that of Subtype 2 (*p* < 0.001, *T* = 2.654) in the cerebellum. In addition, compared with Subtype2, Subtype 1 showed lower stability of dynamic functional architecture (Additional file 1: Fig. S11).

In terms of two sample t-test between healthy controls and ASD subtypes, Subtype 1 showed lower dynamic R-fMRI measures including DC, GSCorr and ReHo (Additional file 1: Fig. S12). In addition, compared with healthy controls, Subtype 1 showed lower stability of dynamic functional architecture, in contrast, Subtype 2 experienced higher stability of dynamic functional architecture (Additional file 1: Fig. S13).

### Improved classification accuracy using SVM

In the classification of healthy controls and all ASD patients, the SVM model obtained a mean classification accuracy of 51.37%. By using HYDRA to divide the ASD into 2 subtypes, the classification accuracies were significantly improved, with a classification accuracy of 68.17% between healthy controls and Subtype 1 and a classification accuracy of 68.69% between healthy controls and Subtype 2. In addition, we calculated the average weight of each ROI in subtype-healthy control classification by linear SVM. We found that the weight ranking of the two subtype classification experiments had a great difference (Additional file 1: Fig. S14).

## Discussion

**Fig. 5:**
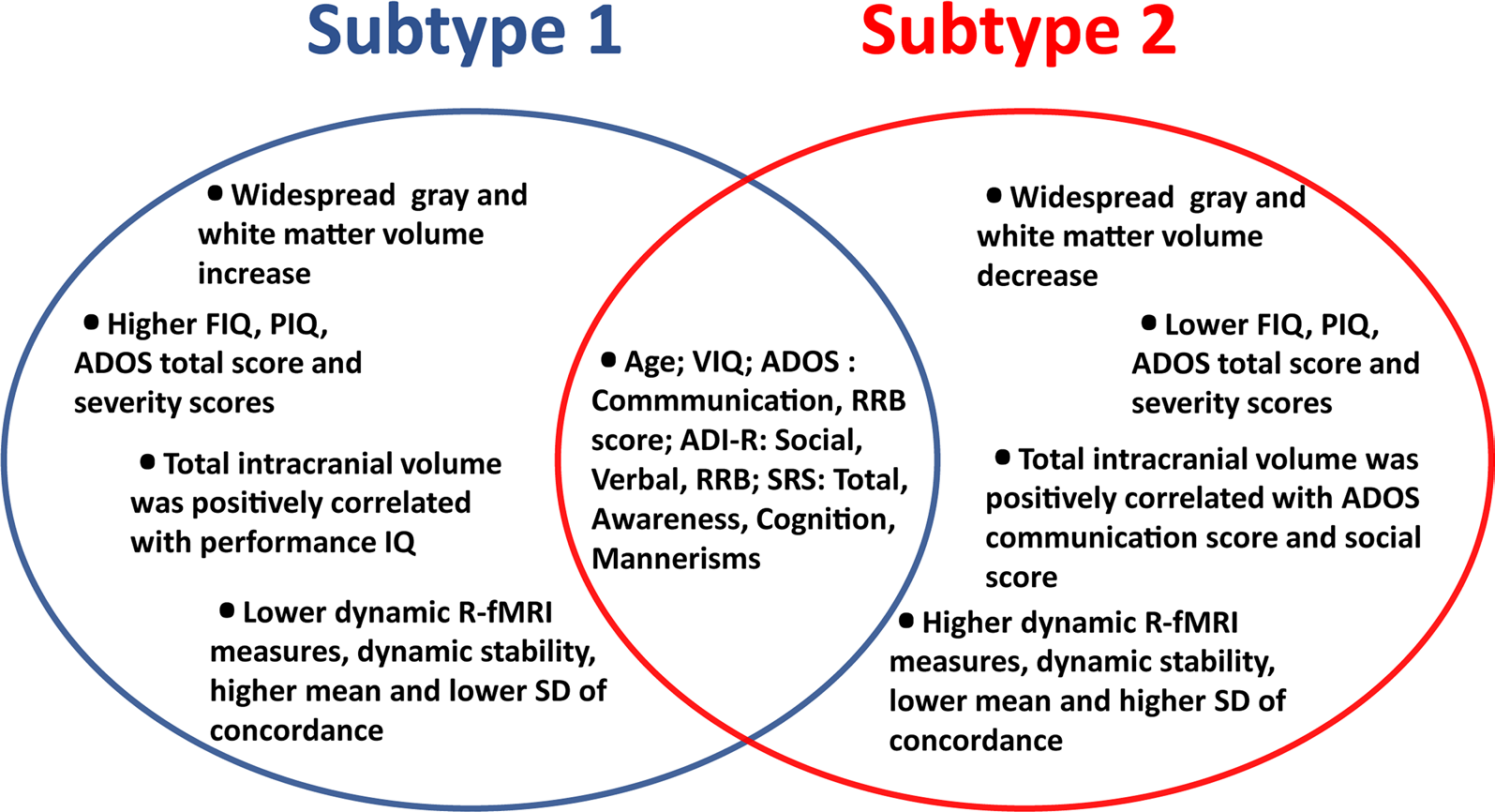
Main findings of similarities and differences between subtype 1 and subtype 2. Abbreviations: FIQ, full-scale IQ; PIQ, performance IQ; VIQ, verbal IQ; ADOS, autism diagnostic observation schedule; R-fMRI, resting-state functional magnetic resonance imaging; SD, standard deviation; RRB, stereotyped behaviors and restricted interests; ADI-R, autism diagnostic interview revised; SRS, Social Responsiveness Scale

The current ASD diagnostic system assigns a single, behaviorally-defined label to a population composed of different subgroups that may have different etiologies [31]. The distinct inter-subject heterogeneity of ASD has been considered to represent one of the most important obstacles for objective diagnosis and optimized treatment [32, 33]. The inter-subject heterogeneity may conceal group level differences in ASD and objectively defining biological subtypes is a crucial step in the future. In this study, an advanced semi-supervised clustering approach was used to subtype male ASD into two neurosubtypes based on regional volumetric profiles from structural MRI data. In accord with the latest knowledge of neuroimaging heterogeneity, our analysis revealed that the subtypes which were not detectible by clinical measures, had brain-behavior and brain functional differences (Fig. 5).

There is a consensus that heterogeneity underlies the neurobiology of ASD [34]. To address the neurobiological heterogeneity of ASD, researchers have begun to apply data-driven strategies to subtype ASD based on neuroimaging features [20]. However, the research on neurosubtypes of ASD have been highly diverse [20]. Simply put, in all studies, it was found that there were 2–4 ASD neurosubtypes [20]. These nascent studies vary in data sources, simple size, sex ratio, methodology, neuroimaging features. In addition, a recent state-of-the-art review on neurosubtypes in ASD has pointed out the necessity for minimizing confounding factors such as head motion-induced artifacts and measurement noise in neuroimaging features [20]. In this study, large multi-site samples are included to ensure the reliability of the research results, and two distinct neurosubtypes of ASD are uncovered. A previous study based on voxel-wise mapping and with similar sample characteristics (sample source, sample size and sex radio) identified three ASD neurosubtypes [17], which was inconsistent with the present findings. One plausible explanation was that the previous study attempted to exclude the influence of IQ in subtyping. On the contrary, we took IQ into consideration in the subtyping of ASD because that IQ would change accordingly due to the process of the disease [5, 35], we did not treat IQ as a confounding factor.

Previous studies on data-driven neurosubtyping of ASD have mainly used unsupervised clustering approaches such as k-means clustering or hierarchical clustering [17–19]. K-means clustering requires specifying cluster numbers beforehand, while hierarchical clustering may potentially lead to suboptimal findings [20]. In order to overcome the high heterogeneity of ASD, we used a recently-proposed semi-supervised machine learning method named HYDRA [21]. HYDRA develops a novel non-linear learning algorithm for integrated binary classification and subpopulation clustering, it uniquely excavates cluster illness effects by modelling differences from healthy controls instead of clustering patients directly [21, 22]. Unsupervised clustering algorithms like k-means cluster patients according to the similarity of patients, which is easy to confound inter- individual diversity and variability irrelevant to disease [21]. HYDRA can identify true disease subtypes by removing the influence of confounding variations introduced by age, sex, scanner variation, ethnicity, and other factors, and can effectively find the optimal number of subtypes by varying the number of hyperplanes [21]. In this study, the two subtypes clustered by HYDRA were robust to split sample experiments and leave-one-site-out experiments, which proved that our neurosubtypes had high cross-site reproducibility.

For neuroimaging features used in ASD neurosubtyping studies, most of the previous studies have focused on a single neuroimaging modality (structural features such as cortical thickness, geodesic distance, intensity contrast and surface area; or functional features like functional connectivity and brain network), primarily structural or functional MRI [17–20]. However, in neuroimaging field, functional measures have faced with issues of moderate reliability [36]. Different from functional measures, the current study selected reliable features from structural MRI. Furthermore, other data modalities including clinical information and dynamic R-fMRI measures were used to validate the rationality of the resultant two neuroanatomical subtypes. To address confounding factors such as measurement noise and head motion-induced noise, a strict and objective structural image quality control threshold is applied in the current study. Head motion-induced artifacts are addressed using strict excluding threshold (sub-voxel level threshold) in the fMRI data processing.

Heterogeneity of ASD is originated from genetic variation, since high-throughput genomic methods revealed substantial variations of genetic architecture in ASD [34]. However, subtyping of ASD based on genetic approaches is challenging [20]. On the contrary, neuroimaging stands a chance to bridge from micro underlying mechanisms to macro clinical phenotype. Since previous study, a consensus has been reached that brain structural heterogeneity might be one characteristic of ASD [9]. In fact, brain structural heterogeneity accompanies ASD patients through neural development. Some children with ASD show early onset who have signs of developmental delays within the first 18 months of life. However, 25–40% of children develop normally until 18–24 months, when they degenerate into ASD [37]. During early and late childhood, different patterns such as early overgrowth, slowed down or arrested growth of the brain may be manifested in ASD children. The differences in brain growth may account for brain structural heterogeneity of ASD [38, 39]. In addition, the different ASD subtypes may be associated with differential neurodevelopment disruptions driven by both the variation of gene copy number and environmental toxins, as a recent study estimated that approximately 20% of individuals with ASD presented with de novo genetic mutations [40], and the de novo genes play a role in regulating synaptic development, neuron motility and axon guidance [41]. In this sense, the present two neurosubtypes of ASD take the first step towards understanding the neurobiology of ASD. A remaining challenge is to associate macroscale brain structural heterogeneity to microscale underlying mechanisms.

Previous studies have shown that the abnormal brain enlargement observed in ASD during early childhood is disproportionately accounted for by increased white matter, not gray matter [42], since some studies have shown greater increases in white matter than gray matter in young children [43, 44]. On the contrary, recent studies have shown an opposite trend of developmental differences observed during early brain maturation (later childhood and adolescence) in ASD which seems to be dominated by an accelerated age-related decline in gray matter volume [45, 46]. Notably, conventional case–control analyses may average across important inter-individual variability, ultimately yielding an average state, leading to the result biased to one side (an overall increase or decrease). This may be the reason for the obvious inconsistency in the traditional group comparison [47]. The present results revealed two different neuroanatomical patterns across a large sample of ASD subjects. Specifically, we found completely opposite patterns of gray and white matter distribution in the two subtypes of ASD patients but there was no significant difference in age between the two subtypes. In line with the above view, we suggest that gray matter or white matter may decrease in some subgroups and increase in different subgroups during early brain maturation in ASD and the brain abnormal development of ASD may potentially cause different neuroanatomical subtypes observed in current study.

In the current study, we found that the two neuroanatomically subtypes differed in clinical measures. Specifically, there were significant differences in full-scale IQ and performance IQ between the two ASD subtypes. And the subtype with higher IQ exhibited severe clinical symptoms, while the subtype with lower IQ exhibited moderate clinical symptoms. This seems paradoxical considering that ASD is characterized by intellectual disability. However, this paradox may be resolved by a series of genetic findings that alleles for ASD overlap broadly with alleles for high intelligence [48, 49]. In addition, the paradox is supported by the neuroanatomical result that the two ASD subtypes experienced distinct gray and white matter volumetric patterns. Specifically, a previous study has shown that there is a significant positive correlation between the volume of gray matter and white matter and IQ [50]. Our results are consistent with previous findings, compared with subtype 2, subtype 1 has higher gray matter volume and higher IQ. One possible explanation is that the high heterogeneity of ASD development changes the brain structure and further affects the intellectual development of ASD patients. This partly explains the variances in nonverbal IQ in ASD patients [5]. Previous studies suggested that neuroanatomy was related to cognition [51] and clinical symptomatology of ASD patients [40]. In fact, the wide clinical heterogeneity among subjects with ASD hinders the diagnosis and treatment of ASD [52, 53]. Our results demonstrate that the brain-behavior relationships could be built across two subtypes, this brain-behavior relationships have the potential to improve the precision diagnosis and treatment of ASD patients, which was also supported by the improved classification accuracies of the machine learning model.

The functional organization of the brain changes dynamically over sessions, even during rest [54]. Some studies have pointed out that the human brain responds to internal or external stimuli through dynamic integration and adjustment on various time scales [55]. For this reason, we performed dynamic R-fMRI analysis and found different dynamic functional patterns in the two subtypes. Specifically, two subtypes showed significantly different dynamics and functional stability in the cerebellum. More importantly, differences in cerebellar structure and function are among inconsistent findings in patients with ASD, suggesting that cerebellar dysfunction may be important in the heterogeneity of ASD [56–58]. Therefore, our findings in the cerebellum may reconcile the inconsistent findings in previous studies [56–58] and may imply that two different etiologies are between the two subtypes. In addition, we found significantly different abilities of functional integration in the two subtypes through the concordance among R-fMRI measures. Combined with the differences of cognitive function between the two anatomical subtypes of ASD, we speculate that the functional stability of ASD patients may be related to cognitive function, since various complex cognitive functions require the brain to coordinate information from multiple patterns over time [54, 59].

### Limitations

There are several limitations in the present study. First, the present study excluded female subjects and left-handed subjects, which limited the ability to investigate the associations between these factors and the heterogeneity of ASD. To overcome this issue, a larger dataset which contains female and left-handed subjects are needed. Furthermore, other distinctions within these subtypes may be discovered, resulting in more fine-grained parsing of heterogeneity by larger samples. Second, while the large and multisite sample in this study is an advantage, it also reduces the depth of clinical phenotyping due to different clinical cognitive scales used across sites. Third, while cross-sectional profiles have clear subtyping value, they only capture a single point in time. Enriched profiles can be derived from longitudinal data, which allows to explore changes of ASD subtypes over time. Finally, despite the advantages of reporting effect sizes and p-value for comparisons of independently collected samples, and the usage of strict multiple comparison correction methods, it is worth mentioning that they are not without limitations, and therefore, the present results should be carefully interpreted.

## Conclusions

To sum up, through strict data inclusion criteria, large sample, and multi-site individuals with ASD were obtained. We used an advanced semi-supervised machine learning method to subtype ASD. Two distinct and highly reproducible neuroanatomical subtypes of ASD were found. Moreover, ASD patients belonging to different subtypes also showed different clinical and dynamic R-fMRI measures. This work suggests the existence of ASD subtypes at the neuroanatomical and functional level, and reveals the potential of such findings not only in improving our understanding of the mechanism of abnormal brain development in ASD, but also in the development of potential stable brain-behavior relationships of the disorder. With future research, these subtypes could potentially be used to improve clinical decision-making process and optimize treatment in the future.

## Supplementary Information

**Additional file 1**. **Table S1.** Quality control metrics for the enrolled subjects. **Table S2.** Lists number of ASD subjects with available clinical measures. **Table S3.** List of brain regions in the Neuromorphometrics atlas used as features in HYDRA. **Table S4.** Detailed statistical analysis of age between HC and ASD in each site and full cohort. **Table S5**. Clinical profiles between subgroups across the 7 sites. **Fig. S1.** The image quality measures describe the properties of the image. **Fig. S2.** Examples of different quality ratings for the original T1 images (left) and segmented images (right). **Fig. S3.** Schematic illustration of the HYDRA method. Controls (denoted by blue squares) are separated from the patients (denoted by red triangles) using a convex polytope decision boundary. Solid lines correspond to the classifier, dashed lines indicate the margin while highlighted linear segments define the separating convex polytope. **Fig. S4.** Cross-validated stability of ASD subtypes: Adjusted Rand Index (ARI) vs. number of subtypes (K) indicating highest reproducibility when K = 2. **Fig. S5.** Volume difference in gray matter (A) and white matter (B) volume between healthy control (HC) (n = 257) and ASD (n = 221) by standard case-control comparison. Effect size (Cohen’s d) maps were generated from regional volumetric maps masked by the set of regions that showed statistically significant differences (P_FDR_ < 0.05) in the MIDAS analysis. **Fig. S6.** GM volumetric differences between each subtype and HC for K = 2 in Split 1 (left column) and Split 2 (right column). **Fig. S7.** WM volumetric differences between each subtype and HC for K = 2 in Split 1 (left column) and Split 2 (right column). **Fig. S8.** The number of overlaps assigned to the same ASD subtype in the leave-one-site-out strategy. **Fig. S9.** Temporal SD of dynamic R-fMRI measures differences between ASD 1 and ASD 2 using the two-sample t-tests for (a) DC, (b) GSCorr and (c) ReHo (GRF, voxel-level p < 0.001, cluster-level p < 0.01, two-tailed).** Fig. S10.** (a) Two sample t-test of the mean of concordance among R-fMRI indices between ASD 1 and ASD 2; (b) Two sample t-test of the SD of concordance among R-fMRI indices between ASD 1 and ASD 2. Of note, the demonstrated mean/SD were fitted values with the effect of head motion, age and site regressed out. **Fig. S11.** Temporal SD of dynamic stability measures differences between ASD 1 and ASD 2 using two-sample t-test (GRF correction at voxel-level p < 0.001, cluster-level p < 0.01, two-tailed). **Fig. S12.** Temporal SD of dynamic R-fMRI measures differences between healthy controls and ASD 1 using the two-sample t-tests for (a) DC, (b) GSCorr and (c) ReHo (GRF, voxel-level p < 0.001, cluster-level p < 0.01, two-tailed). **Fig. S13.** (a) Temporal SD of dynamic stability measures differences between healthy controls and ASD 1 using two-sample t-test (GRF correction at voxel-level p < 0.001, cluster-level p < 0.01, two-tailed). (b) Temporal SD of dynamic stability measures differences between healthy controls and ASD 2 using two-sample t-test (GRF correction at voxel-level p < 0.001, cluster-level p < 0.01, two-tailed). **Fig. S14.** Average weight ranking differences of ROI in the subtype-healthy control classification. Subtype1-healthy controls (Left) and subtype2-healthy controls (Right). The average weight of the top 40 ROIs is shown here.

## Data Availability

Data were provided by the Autism Brain Imaging Data Exchange repository in the international neuroimaging data-sharing initiative datasets (http://fcon_1000.projects.nitrc.org/indi/abide/).

## Abbreviations

ASD: Autism spectrum disorder
IQ: Intelligence quotient
RRB: Restricted repetitive behavior
VBM: Voxel-based morphometry
ROI: Region of interest
CSF: Cerebrospinal fluid
MRI: Magnetic resonance imaging
HYDRA: Heterogeneity through discriminative analysis
R-fMRI: Resting-state functional magnetic resonance imaging
ABIDE: Autism Brain Image Data Exchange database
IQR: Image Quality Rate
NCR: Noise Contrast Ratio
ICR: Inhomogeneity Contrast Ratio
RES: RMS resolution
NYU: New York University Medical Center
KKI: Kennedy Krieger Institute
MNI: Montreal Neurological Institute
FWHM: Full width at half maximum
ARI: Adjusted rand index
MIDAS: Multivariate discriminative statistical mapping
FDR: False discovery rate
ADOS: Autism Diagnostic Observation Schedule
SVM: Support vector machine
ReHo: Regional homogeneity
VMHC: Voxel-mirrored homotopic connectivity
DC: Degree centrality
GSCorr: Global signal correlation
SD: Standard deviation
KCC: Kendall’s coefficient of concordance
GRF: Gaussian Random Field
