# Supplementary material for: Two neuroanatomical subtypes of males with autism spectrum disorder revealed using semi-supervised machine learning
Source: Mol Autism. 2022 Feb 23;13:9. doi: 10.1186/s13229-022-00489-3 (PMC8867630; doi:10.1186/s13229-022-00489-3)
Supplement: Supplementary file 1 — Additional file 1. Table S1. Quality control metrics for the enrolled subjects. Table S2. Lists number of ASD subjects with available clinical measures. Table S3. List of brain regions in the Neuromorphometrics atlas used as features in HYDRA. Table S4. Detailed statistical analysis of age between HC and ASD in each site and full cohort. Table S5. Clinical profiles between subgroups across the 7 sites. Fig. S1. The image quality measures describe the properties of the image. Fig. S2. Examples of different quality ratings for the original T1 images (left) and segmented images (right). Fig. S3. Schematic illustration of the HYDRA method. Controls (denoted by blue squares) are separated from the patients (denoted by red triangles) using a convex polytope decision boundary. Solid lines correspond to the classifier, dashed lines indicate the margin while highlighted linear segments define the separating convex polytope. Fig. S4. Cross-validated stability of ASD subtypes: Adjusted Rand Index (ARI) vs. number of subtypes (K) indicating highest reproducibility when K = 2. Fig. S5. Volume difference in gray matter (A) and white matter (B) volume between healthy control (HC) (n = 257) and ASD (n = 221) by standard case-control comparison. Effect size (Cohen’s d) maps were generated from regional volumetric maps masked by the set of regions that showed statistically significant differences (PFDR < 0.05) in the MIDAS analysis. Fig. S6. GM volumetric differences between each subtype and HC for K = 2 in Split 1 (left column) and Split 2 (right column). Fig. S7. WM volumetric differences between each subtype and HC for K = 2 in Split 1 (left column) and Split 2 (right column). Fig. S8. The number of overlaps assigned to the same ASD subtype in the leave-one-site-out strategy. Fig. S9. Temporal SD of dynamic R-fMRI measures differences between ASD 1 and ASD 2 using the two-sample t-tests for (a) DC, (b) GSCorr and (c) ReHo (GRF, voxel-level p < 0.001, cluster-level p < 0.01, two-ta [file 13229_2022_489_MOESM1_ESM.docx]

**Supplementary Table S1. Quality control metrics for the enrolled subjects.**

|  | **Subtype 1**  **(n = 115)** | **Subtype 2**  **(n = 106)** | **HC**  **(n = 257)** | **P value ^a^** | **P value ^b^** | **P value ^c^** |
| --- | --- | --- | --- | --- | --- | --- |
| **RES** | 1.96 ± 0.187 | 1.94 ± 0.207 | 1.96 ± 0.176 | 0.587 | 0.652 | 0.273 |
| **NCR** | 1.88 ± 0.368 | 1.88 ± 0.416 | 1.83 ± 0.367 | 0.980 | 0.265 | 0.312 |
| **ICR** | 2.04 ± 0.324 | 1.95 ± 0.379 | 1.98 ± 0.361 | 0.058 | 0.146 | 0.435 |
| **IQR** | 1.95 ± 0.249 | 1.94 ± 0.293 | 1.95 ± 0.241 | 0.954 | 0.656 | 0.734 |

The p values were calculated using a two-sample t-test (^a^, Subtype1 vs Subtype2; ^b^, HC vs Subtype1; ^c^, HC vs Subtype2).

Abbreviations: RES, RMS resolution; NCR, noise contrast ratio; ICR, inhomogeneity contrast ratio; IQR, image quality rate.

**Supplementary Table S2. The number of ASD patients with available clinical measures.**

|  | **Site 1**  **(GU)** |  | **Site 2**  **(KKI)** |  | **Site 3**  **(OHSU)** |  | **Site 4**  **(PITT)** |  | **Site 5**  **(TRINITY)** |  | **Site 6**  **(UCLA)** |  | **Site 7**  **(USM)** | **ALL** |
| --- | --- | --- | --- | --- | --- | --- | --- | --- | --- | --- | --- | --- | --- | --- |
| **IQ** |  | | | | | | | | | | | | | |
| **FIQ** | n = 25 |  | n = 28 |  | n = 35 |  | n = 20 |  | n = 22 |  | n = 38 |  | n = 53 | n = 221 |
| **VIQ** | n = 25 |  | n = 19 |  | **—** |  | n = 20 |  | n = 22 |  | n = 37 |  | n = 53 | n = 176 |
| **PIQ** | n = 20 |  | n = 19 |  | **—** |  | n = 20 |  | n = 21 |  | n = 37 |  | n = 53 | n = 170 |
| **ADOS** |  | | | | | | | | | | | | |  |
| **Total** | n = 19 |  | n = 18 |  | n = 10 |  | n = 18 |  | n = 22 |  | n = 36 |  | n = 50 | n = 173 |
| **Comm** | n = 19 |  | n = 18 |  | n = 10 |  | n = 18 |  | **—** |  | n = 36 |  | n = 50 | n = 151 |
| **Social** | n = 19 |  | n = 18 |  | n = 10 |  | n = 18 |  | **—** |  | n = 36 |  | n = 50 | n = 151 |
| **RRB** | n = 19 |  | n = 18 |  | n = 10 |  | n = 18 |  | **—** |  | n = 36 |  | n = 48 | n = 149 |
| **Severity** | n = 6 |  | n = 19 |  | n = 25 |  | **—** |  | **—** |  | n = 33 |  | n = 14 | n = 97 |
| **ADI-R** |  | | | | | | | | | | | | | |
| **Social** | n = 25 |  | n = 28 |  | n = 35 |  | n = 18 |  | n = 22 |  | n = 31 |  | **—** | n = 159 |
| **Verbal** | n = 25 |  | n = 28 |  | n = 35 |  | n = 18 |  | n = 22 |  | n = 31 |  | **—** | n = 159 |
| **RRB** | n = 25 |  | n = 28 |  | n = 35 |  | n = 18 |  | n = 22 |  | n = 31 |  | **—** | n = 159 |
| **SRS** |  | | | | | | | | | | | | | |
| **Total** | n = 25 |  | n = 19 |  | n = 24 |  | **—** |  | **—** |  | **—** |  | n = 53 | n = 121 |
| **Aware** | n = 25 |  | n = 19 |  | n = 24 |  | **—** |  | **—** |  | **—** |  | n = 7 | n = 75 |
| **Cognit** | n = 25 |  | n = 19 |  | n = 24 |  | **—** |  | **—** |  | **—** |  | n = 7 | n = 75 |
| **Comm** | n = 25 |  | n = 19 |  | n = 24 |  | **—** |  | **—** |  | **—** |  | n = 7 | n = 75 |
| **Motiva** | n = 25 |  | n = 19 |  | n = 24 |  | **—** |  | **—** |  | **—** |  | n = 7 | n = 75 |
| **Manner** | n = 25 |  | n = 19 |  | n = 24 |  | **—** |  | **—** |  | **—** |  | n = 7 | n = 75 |

Abbreviations: ASD, autism spectrum disorder; GU, Georgetown University; KKI, Kennedy Krieger Institute; OHSU, Oregon Health and Science University; PITT, University of Pittsburgh School of Medicine; TRINITY, Trinity Centre for Health Sciences; UCLA, University of California, Los Angeles; USM, University of Utah School of Medicine; IQ, intelligence quotient; FIQ, full-scale IQ; VIQ, verbal IQ; PIQ, performance IQ; N, number; ADOS, autism diagnostic observation schedule; Comm, communication, RRB, stereotyped behaviors and restricted interests.; ADOS Total = social + communication; ADI-R, autism diagnostic interview revised; SRS, social responsiveness scale; Aware, awareness; Cognit, cognition; Motiva, motivation; Manner, mannerisms; SRS Total = awareness + cognition + communication + motivation + mannerisms.

**Supplementary Table S3.** **List of brain regions in the Neuromorphometrics atlas used as features in HYDRA.**

| ID | Brain regions | ID | Brain regions | ID | Brain regions |
| --- | --- | --- | --- | --- | --- |
| 1 | Left Third Ventricle | 51 | Left Angular Gyrus | 101 | Left Inferior Frontal Orbital Gyrus |
| 2 | Right Third Ventricle | 52 | Right Angular Gyrus | 102 | Right Inferior Frontal Orbital Gyrus |
| 3 | Left Fourth Ventricle | 53 | Left Calcarine and Cerebrum | 103 | Left Posterior Cingulate Gyrus |
| 4 | Right Fourth Ventricle | 54 | Right Calcarine and Cerebrum | 104 | Right Posterior Cingulate Gyrus |
| 5 | Left Accumbens | 55 | Left Central Operculum | 105 | Left Precuneus |
| 6 | Right Accumbens | 56 | Right Central Operculum | 106 | Right Precuneus |
| 7 | Left Amygdala | 57 | Left Cuneus | 107 | Left Parahippocampus Gyrus |
| 8 | Right Amygdala | 58 | Right Cuneus | 108 | Right Parahippocampus Gyrus |
| 9 | Left Brainstem | 59 | Left Entorhinal Area | 109 | Left Posterior Insula |
| 10 | Right Brainstem | 60 | Right Entorhinal Area | 110 | Right Posterior Insula |
| 11 | Left Caudate | 61 | Left Frontal Operculum | 111 | Left Parietal Operculum |
| 12 | Right Caudate | 62 | Right Frontal Operculum | 112 | Right Parietal Operculum |
| 13 | Left Exterior Cerebellum | 63 | Left Frontal Pole | 113 | Left Postcentral Gyrus |
| 14 | Right Exterior Cerebellum | 64 | Right Frontal Pole | 114 | Right Postcentral Gyrus |
| 15 | Left Cerebellum White Matter | 65 | Left Fusiform Gyrus | 115 | Left Posterior Orbital Gyrus |
| 16 | Right Cerebellum White Matter | 66 | Right Fusiform Gyrus | 116 | Right Posterior Orbital Gyrus |
| 17 | Left Cerebral White Matter | 67 | Left Gyrus Rectus | 117 | Left Planum Polare |
| 18 | Right Cerebral White Matter | 68 | Right Gyrus Rectus | 118 | Right Planum Polare |
| 19 | Left CSF | 69 | Left Inferior Occipital Gyrus | 119 | Left Precentral Gyrus |
| 20 | Right CSF | 70 | Right Inferior Occipital Gyrus | 120 | Right Precentral Gyrus |
| 21 | Left Hippocampus | 71 | Left Inferior Temporal Gyrus | 121 | Left Temporal |
| 22 | Right Hippocampus | 72 | Right Inferior Temporal Gyrus | 122 | Right Temporal |
| 23 | Left Inferior Lateral Ventricle | 73 | Left Lingual Gyrus | 123 | Left Subcallosal Area |
| 24 | Right Inferior Lateral Ventricle | 74 | Right Lingual Gyrus | 124 | Right Subcallosal Area |
| 25 | Left Lateral Ventricle | 75 | Left Lateral Orbital Gyrus | 125 | Left Superior Frontal Gyrus |
| 26 | Right Lateral Ventricle | 76 | Right Lateral Orbital Gyrus | 126 | Right Superior Frontal Gyrus |
| 27 | Left Pallidum | 77 | Left Middle Cingulate Gyrus | 127 | Left Cerebrum and Motor |
| 28 | Right Pallidum | 78 | Right Middle Cingulate Gyrus | 128 | Right Cerebrum and Motor |
| 29 | Left Putamen | 79 | Left Medial Frontal Cerebrum | 129 | Left Supramarginal Gyrus |
| 30 | Right Putamen | 80 | Right Medial Frontal Cerebrum | 130 | Right Supramarginal Gyrus |
| 31 | Left Thalamus Proper | 81 | Left Middle Frontal Gyrus | 131 | Left Superior Occipital Gyrus |
| 32 | Right Thalamus Proper | 82 | Right Middle Frontal Gyrus | 132 | Right Superior Occipital Gyrus |
| 33 | Left Ventral Ventricle | 83 | Left Middle Occipital Gyrus | 133 | Left Superior Parietal Lobule |
| 34 | Right Ventral Ventricle | 84 | Right Middle Occipital Gyrus | 134 | Right Superior Parietal Lobule |
| 35 | Left Optic Chiasm | 85 | Left Medial Orbital Gyrus | 135 | Left Superior Temporal Gyrus |
| 36 | Right Optic Chiasm | 86 | Right Medial Orbital Gyrus | 136 | Right Superior Temporal Gyrus |
| 37 | Left Cerebellar Vermal Lobules I-V | 87 | Left Medial Postcentral Gyrus | 137 | Left Temporal Pole |
| 38 | Right Cerebellar Vermal Lobules I-V | 88 | Right Medial Postcentral Gyrus | 138 | Right Temporal Pole |
| 39 | Left Cerebellar Vermal Lobules VI-VII | 89 | Left Medial Precentral Gyrus | 139 | Left Inferior Frontal Angular Gyrus |
| 40 | Right Cerebellar Vermal Lobules VI-VII | 90 | Right Medial Precentral Gyrus | 140 | Right Inferior Frontal Angular Gyrus |
| 41 | Left Cerebellar Vermal Lobules VIII-X | 91 | Left Superior Medial Frontal Gyrus | 141 | Left Temporal Transverse Gyrus |
| 42 | Right Cerebellar Vermal Lobules VIII-X | 92 | Right Superior Medial Frontal Gyrus | 142 | Right Temporal Transverse Gyrus |
| 43 | Left Basal Cerebrum and Forebrain Brain | 93 | Left Middle Temporal Gyrus |  |  |
| 44 | Right Basal Cerebrum and Forebrain Brain | 94 | Right Middle Temporal Gyrus |  |  |
| 45 | Left Anterior Cingulate Gyrus | 95 | Left Occipital Pole |  |  |
| 46 | Right Anterior Cingulate Gyrus | 96 | Right Occipital Pole |  |  |
| 47 | Left Anterior Insula | 97 | Left Occipital Fusiform Gyrus |  |  |
| 48 | Right Anterior Insula | 98 | Right Occipital Fusiform Gyrus |  |  |
| 49 | Left Anterior Orbital Gyrus | 99 | Left Inferior Frontal Gyrus |  |  |
| 50 | Right Anterior Orbital Gyrus | 100 | Right Inferior Frontal Gyrus |  |  |

**Supplementary Table S4. Detailed statistical analysis of age between HC and ASD in each site and full cohort.**

|  | **Full cohort** | **Split 1** | **Split 2** |
| --- | --- | --- | --- |
| **Site 1 (GU)** | P = 0.518 | P = 0.687 | P = 0.051 |
| **Site 2 (KKI)** | P = 0.713 | P = 0.763 | P = 0.439 |
| **Site 3 (OHSU)** | P = 0.002 | P = 0.004 | P = 0.127 |
| **Site 4 (PITT)** | P = 0.794 | P = 0.833 | P = 0.832 |
| **Site 5 (TRINITY)** | P = 0.997 | P = 0.592 | P = 0.617 |
| **Site 6 (UCLA)** | P = 0.761 | P = 0.556 | P = 0.905 |
| **Site 7 (USM)** | P = 0.758 | P = 0.398 | P = 0.486 |
| **All sites** | P = 0.030 | P = 0.115 | P = 0.134 |

Abbreviations: HC, healthy control; ASD, autism spectrum disorder; GU, Georgetown University; KKI, Kennedy Krieger Institute; OHSU, Oregon Health and Science University; PITT, University of Pittsburgh School of Medicine; TRINITY, Trinity Centre for Health Sciences; UCLA, University of California, Los Angeles; USM, University of Utah School of Medicine.

**Supplementary Table S5. Clinical profiles between subgroups across the 7 sites.**

| **P value** | **Site 1**  **(GU)** |  | **Site 2**  **(KKI)** |  | **Site 3**  **(OHSU)** |  | **Site 4**  **(PITT)** |  | **Site 5**  **(TRINITY)** |  | **Site 6**  **(UCLA)** |  | **Site 7**  **(USM)** |
| --- | --- | --- | --- | --- | --- | --- | --- | --- | --- | --- | --- | --- | --- |
| **IQ** |  |  |  |  |  |  |  |  |  |  |  |  |  |
| **FIQ** | 0.027 |  | 0.494 |  | 0.012 |  | 0.891 |  | 0.984 |  | 0.777 |  | 0.003 |
| **VIQ** | 0.415 |  | 0.659 |  | **—** |  | 0.268 |  | 0.671 |  | 0.568 |  | 0.128 |
| **PIQ** | 0.09 |  | 0.19 |  | **—** |  | 0.641 |  | 0.97 |  | 0.446 |  | <0.001 |
| **ADOS** |  | | | | | | | | | | | | |
| **Total** | 0.714 |  | 0.944 |  | 0.43 |  | 0.939 |  | 0.037 |  | 0.066 |  | 0.465 |
| **Comm** | 0.967 |  | 0.294 |  | 0.187 |  | 0.005 |  | **—** |  | 0.546 |  | 0.241 |
| **Social** | 0.643 |  | 0.604 |  | 0.687 |  | 0.26 |  | **—** |  | 0.023 |  | 0.788 |
| **RRB** | 0.576 |  | 0.321 |  | 0.687 |  | 0.571 |  | **—** |  | 0.33 |  | 0.641 |
| **Severity** | 0.275 |  | 0.022 |  | 0.914 |  | **—** |  | **—** |  | 0.022 |  | 0.92 |
| **ADI-R** |  |  |  |  |  |  |  |  |  |  |  |  |  |
| **Social** | 0.261 |  | 0.528 |  | 0.772 |  | 0.939 |  | 0.463 |  | 0.32 |  | **—** |
| **Verbal** | 0.073 |  | 0.149 |  | 0.18 |  | 0.715 |  | 0.192 |  | 0.643 |  | **—** |
| **RRB** | 0.486 |  | 0.413 |  | 0.042 |  | 0.399 |  | 0.802 |  | 0.836 |  | **—** |
| **SRS** |  |  |  |  |  |  |  |  |  |  |  |  |  |
| **Total** | 0.047 |  | 0.957 |  | 0.152 |  | **—** |  | **—** |  | **—** |  | 0.41 |
| **Aware** | 0.149 |  | 0.653 |  | 0.147 |  | **—** |  | **—** |  | **—** |  | 0.723 |
| **Cognit** | 0.113 |  | 0.547 |  | 0.137 |  | **—** |  | **—** |  | **—** |  | 0.819 |
| **Comm** | 0.018 |  | 0.667 |  | 0.099 |  | **—** |  | **—** |  | **—** |  | 0.956 |
| **Motiva** | 0.303 |  | 0.607 |  | 0.075 |  | **—** |  | **—** |  | **—** |  | 0.546 |
| **Manner** | 0.028 |  | 0.526 |  | 0.558 |  | **—** |  | **—** |  | **—** |  | 0.948 |

The p values were calculated using a two-sample t-test.

Abbreviations: ASD, autism spectrum disorder; GU, Georgetown University; KKI, Kennedy Krieger Institute; OHSU, Oregon Health and Science University; PITT, University of Pittsburgh School of Medicine; TRINITY, Trinity Centre for Health Sciences; UCLA, University of California, Los Angeles; USM, University of Utah School of Medicine; IQ, intelligence quotient; FIQ, full-scale IQ; VIQ, verbal IQ; PIQ, performance IQ; N, number; ADOS, autism diagnostic observation schedule; Comm, communication, RRB, stereotyped behaviors and restricted interests.; ADOS Total = social + communication; ADI-R, autism diagnostic interview revised; SRS, social responsiveness scale; Aware, awareness; Cognit, cognition; Motiva, motivation; Manner, mannerisms; SRS Total = awareness + cognition + communication + motivation + mannerisms.

**
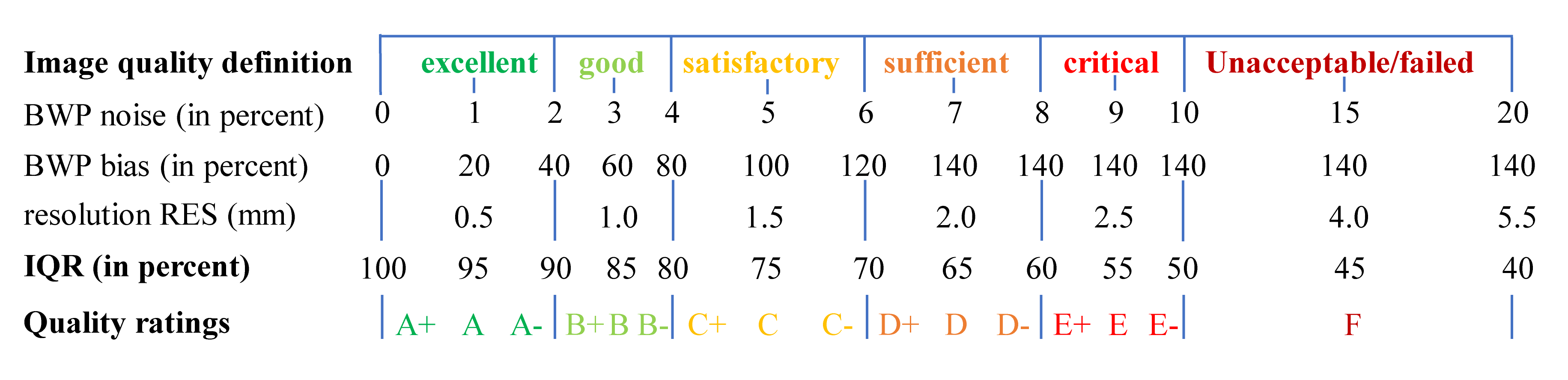
**

**Supplementary Fig. S1.** The image quality measures describe the properties of the image. Supplementary Fig. S2 demonstrates examples of the original T1 structural images and segmented images with different ratings.

**
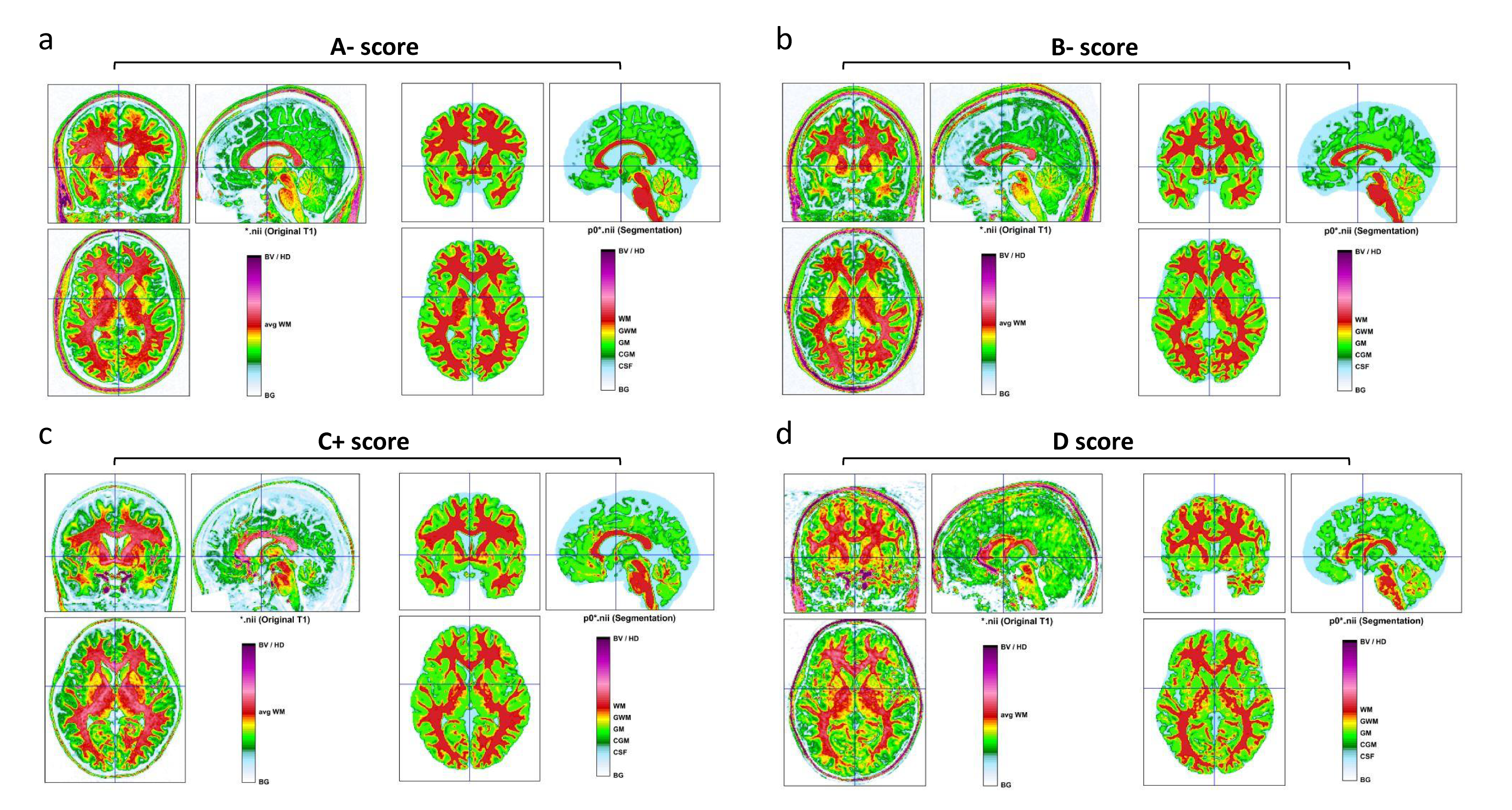
**

**Supplementary Fig. S2.** Examples of different quality ratings for the original T1 images (left) and segmented images (right).

**
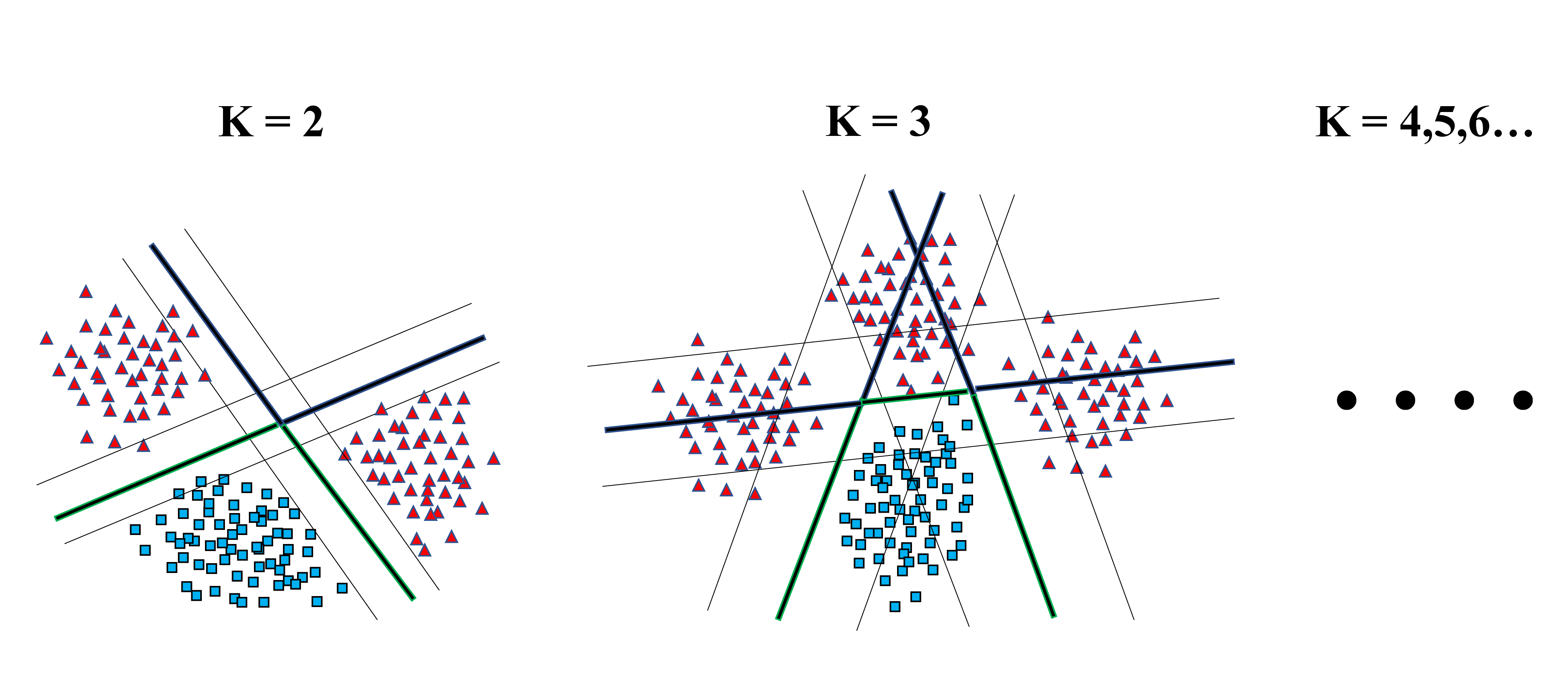
**

**Supplementary Fig. S3.** Schematic illustration of the HYDRA method. Controls (denoted by blue squares) are separated from the patients (denoted by red triangles) using a convex polytope decision boundary. Solid lines correspond to the classifier, dashed lines indicate the margin while highlighted linear segments define the separating convex polytope.

**
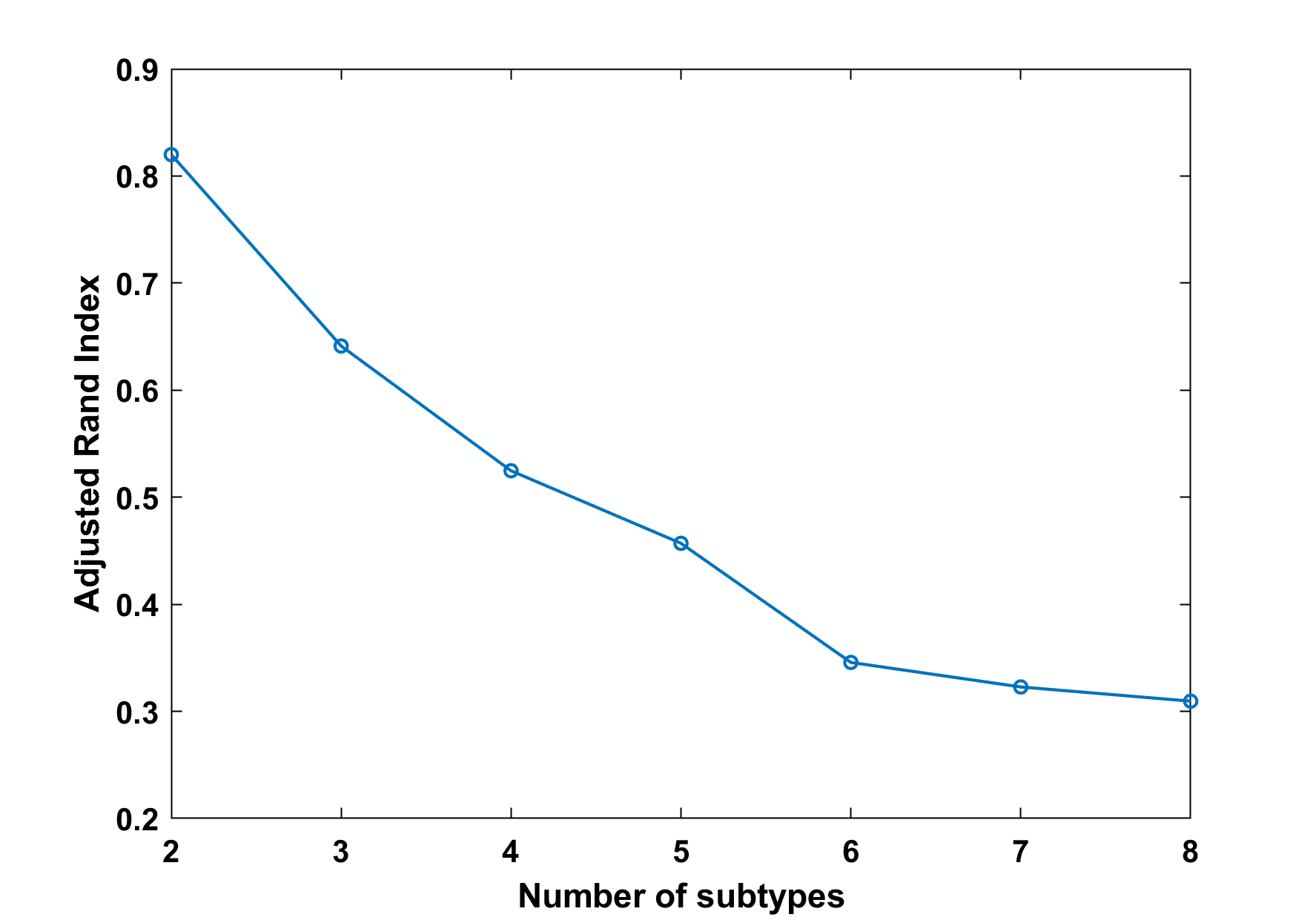
**

**Supplementary Fig. S4.** Cross-validated stability of ASD subtypes: Adjusted Rand Index (ARI) vs. number of subtypes (K) indicating highest reproducibility when K = 2.

**
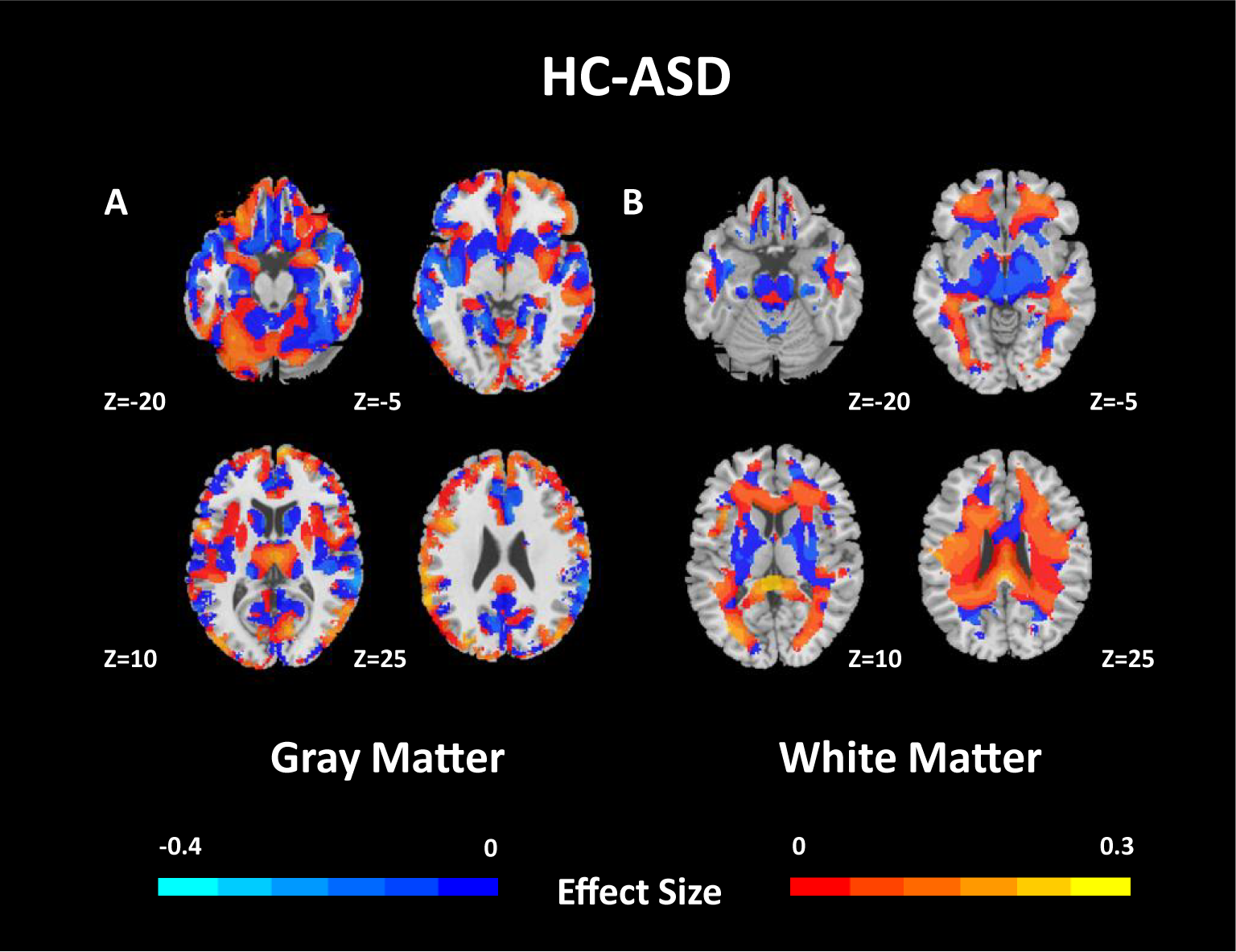
**

**Supplementary Fig. S5.** Volume difference in gray matter (A) and white matter (B) volume between healthy control (HC) (n = 257) and ASD (n = 221) by standard case-control comparison. Effect size (Cohen’s d) maps were generated from regional volumetric maps masked by the set of regions that showed statistically significant differences (P_FDR_ < 0.05) in the MIDAS analysis.

**
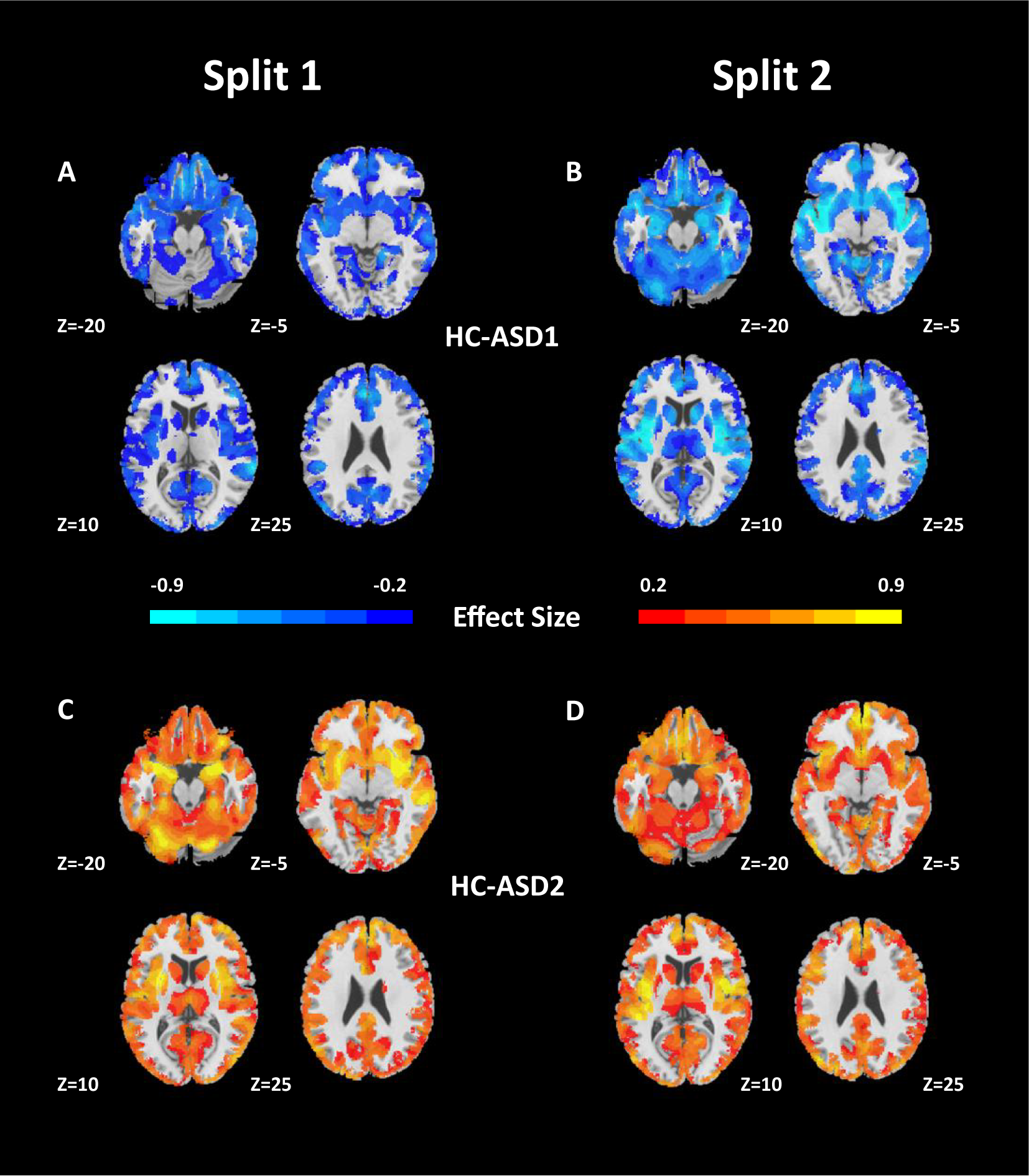
**

**Supplementary Fig. S6.** GM volumetric differences between each subtype and HC for K = 2 in Split 1 (left column) and Split 2 (right column). Abbreviations: HC, healthy control; GM, gray matter.

**
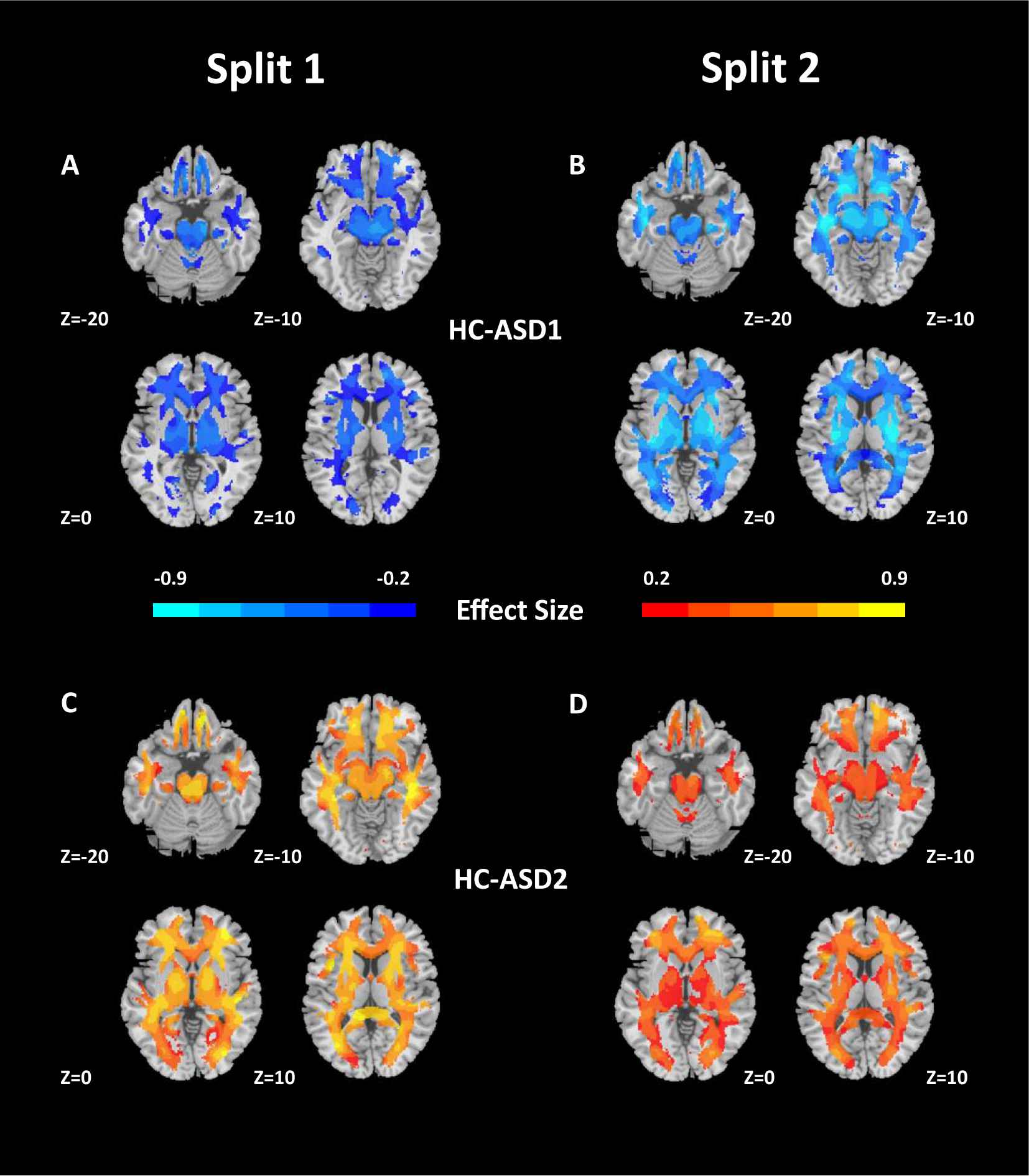
**

**Supplementary Fig. S7.** WM volumetric differences between each subtype and HC for K = 2 in Split 1 (left column) and Split 2 (right column). Abbreviations: HC, healthy control; WM, white matter.


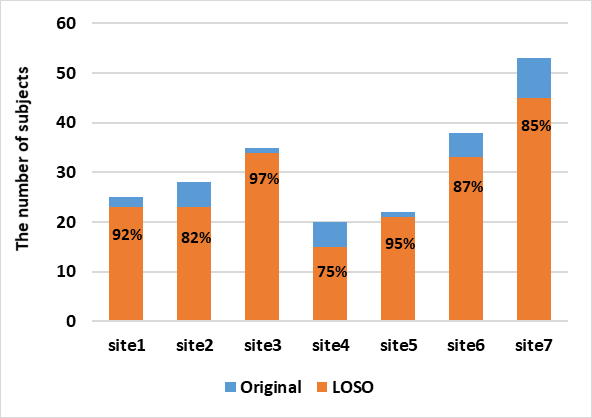


**Supplementary Fig. S8.** The number of overlaps assigned to the same ASD subtype in the leave-one-site-out strategy. Abbreviations: LOSO, leave-one-site-out.


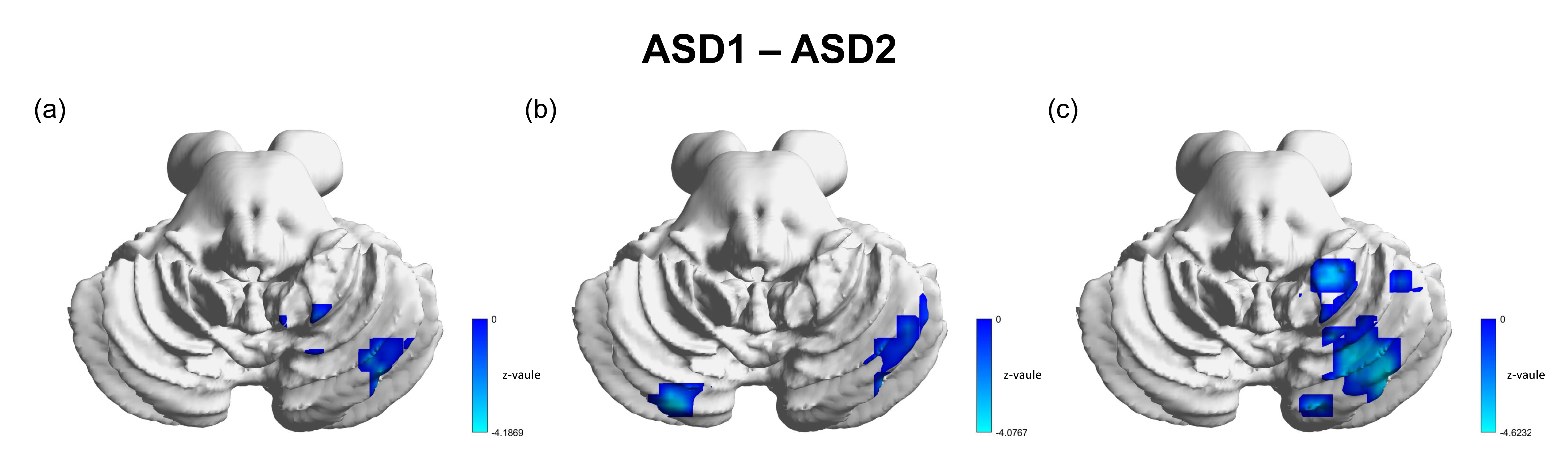


**Supplementary Fig. S9.** Temporal SD of dynamic R-fMRI measures differences between ASD 1 and ASD 2 using the two-sample t-tests for (a) DC, (b) GSCorr and (c) ReHo (GRF, voxel-level p < 0.001, cluster-level p < 0.01, two-tailed). Abbreviations: SD, standard deviation; DC, degree centrality; GSCorr, global signal correlation; ReHo, regional homogeneity; GRF, Gaussian Random Field;


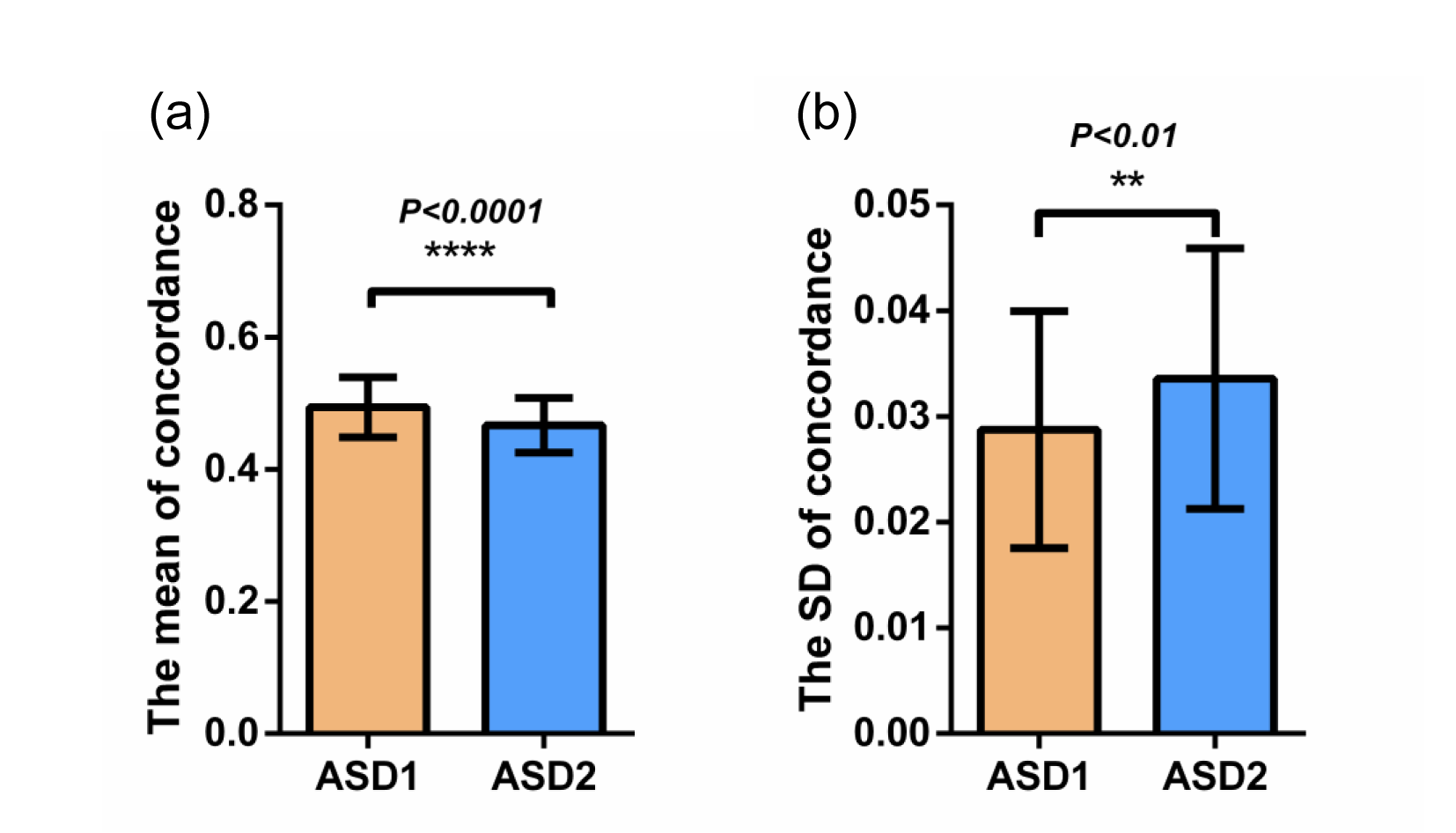


**Supplementary Fig. S10.** (a) Two sample t-test of the mean of concordance among R-fMRI indices between ASD 1 and ASD 2; (b) Two sample t-test of the SD of concordance among R-fMRI indices between ASD 1 and ASD 2. Of note, the demonstrated mean/SD were fitted values with the effect of head motion, age and site regressed out.


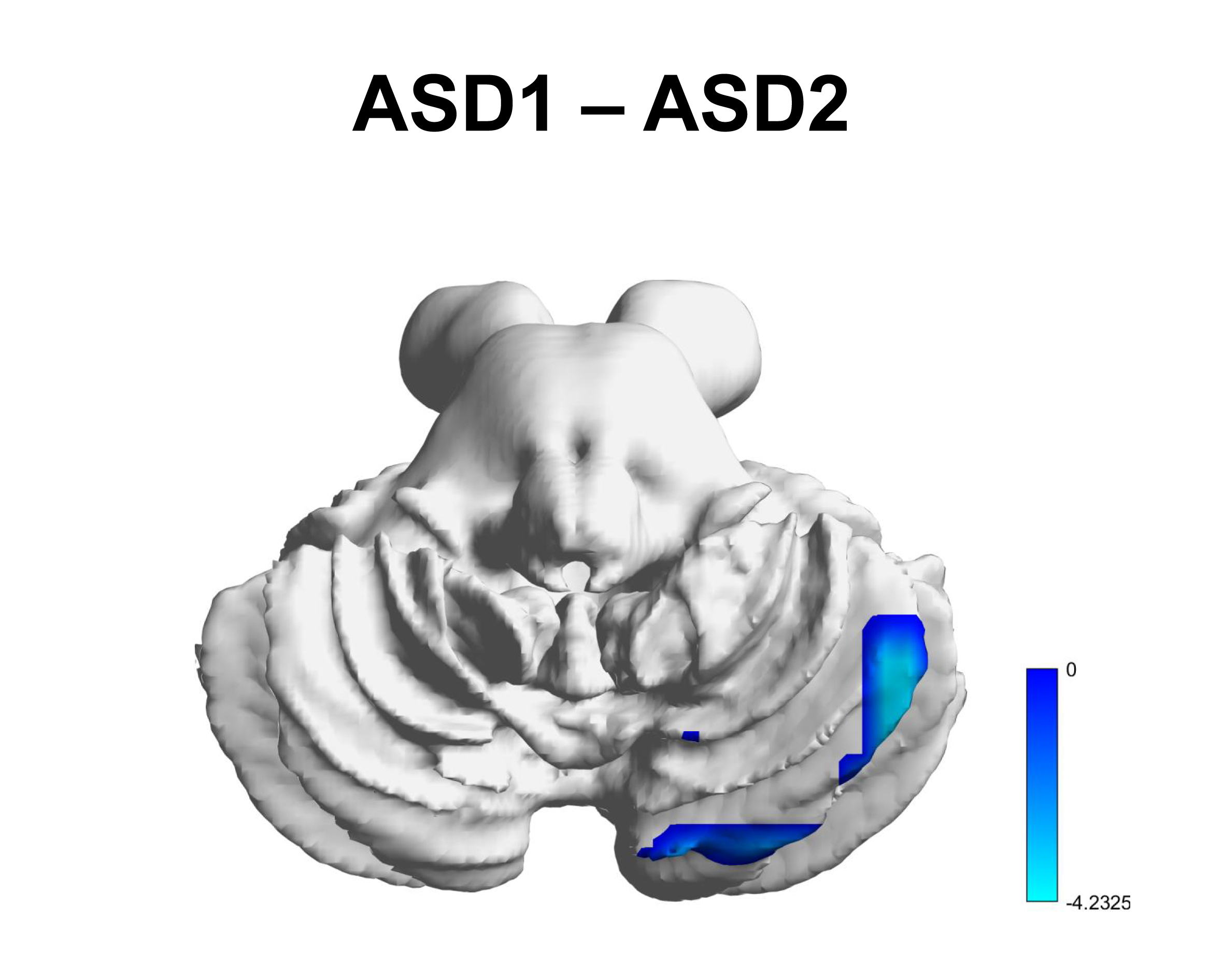


**Supplementary Fig. S11.** Temporal SD of dynamic stability measures differences between ASD 1 and ASD 2 using two-sample t-test (GRF correction at voxel-level p < 0.001, cluster-level p < 0.01, two-tailed). Abbreviations: GRF, Gaussian Random Field;


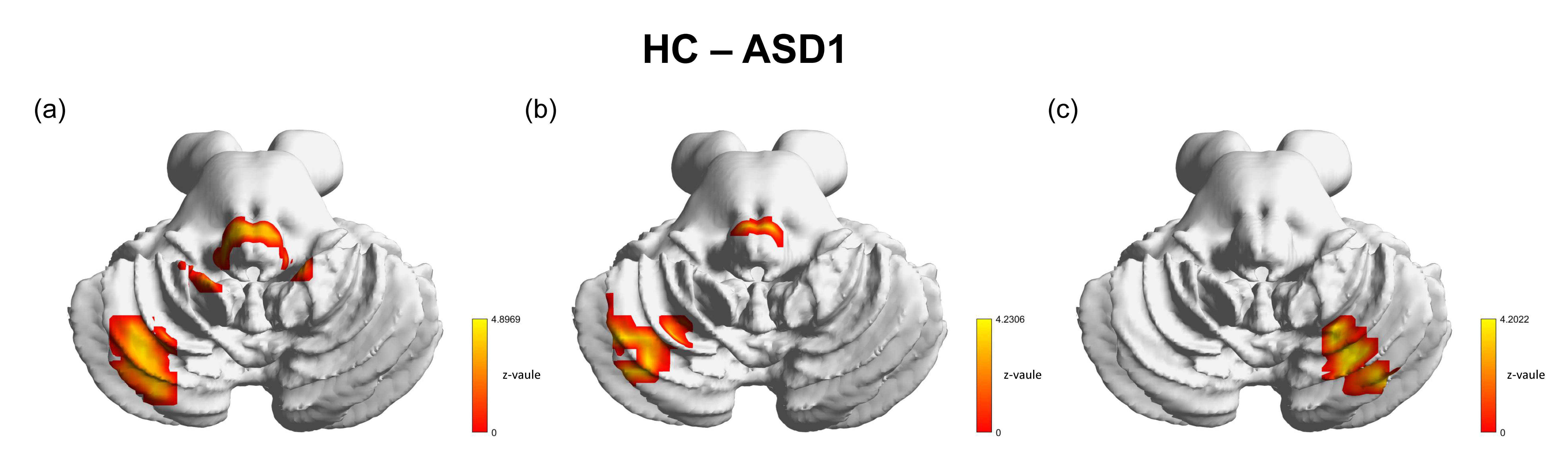


**Supplementary Fig. S12.** Temporal SD of dynamic R-fMRI measures differences between healthy controls and ASD 1 using the two-sample t-tests for (a) DC, (b) GSCorr and (c) ReHo (GRF, voxel-level p < 0.001, cluster-level p < 0.01, two-tailed). Abbreviations: SD, standard deviation; DC, degree centrality; GSCorr, global signal correlation; ReHo, regional homogeneity; GRF, Gaussian Random Field;


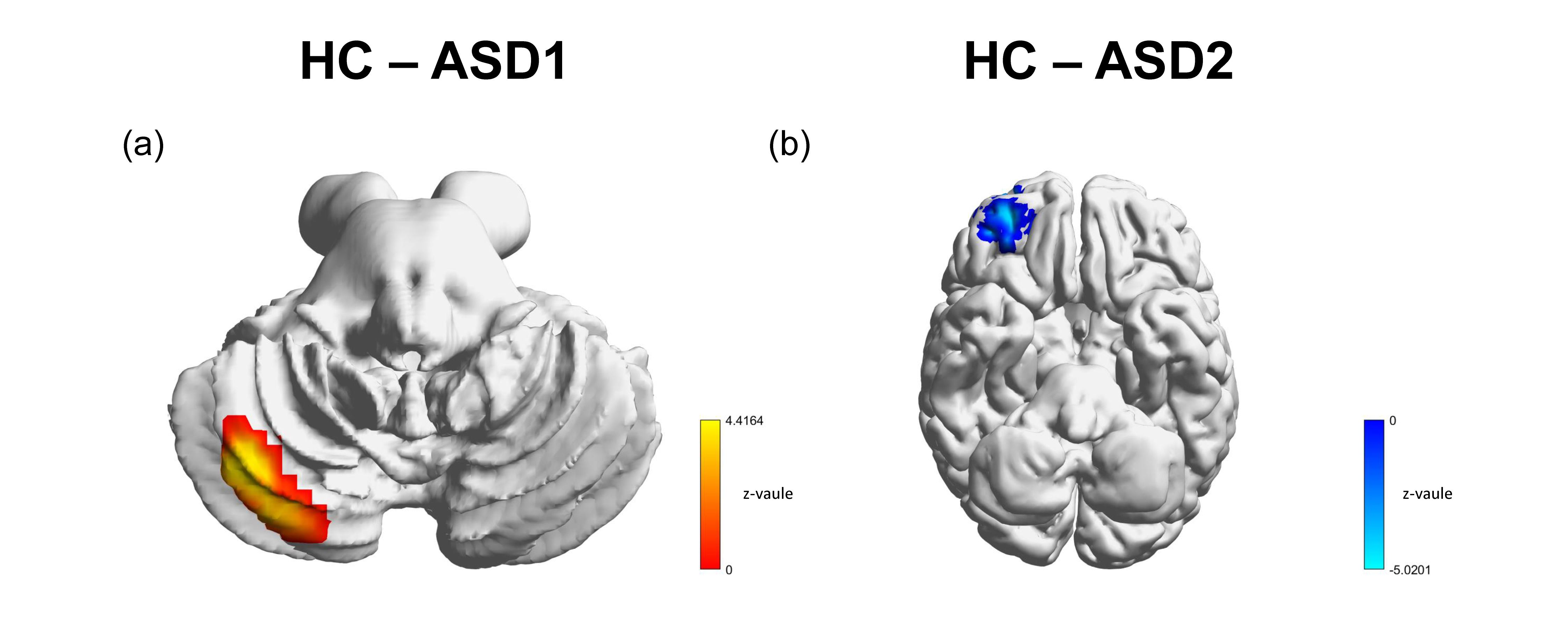


**Supplementary Fig. S13.** (a) Temporal SD of dynamic stability measures differences between healthy controls and ASD 1 using two-sample t-test (GRF correction at voxel-level p < 0.001, cluster-level p < 0.01, two-tailed). (b) Temporal SD of dynamic stability measures differences between healthy controls and ASD 2 using two-sample t-test (GRF correction at voxel-level p < 0.001, cluster-level p < 0.01, two-tailed). Abbreviations: GRF, Gaussian Random Field;


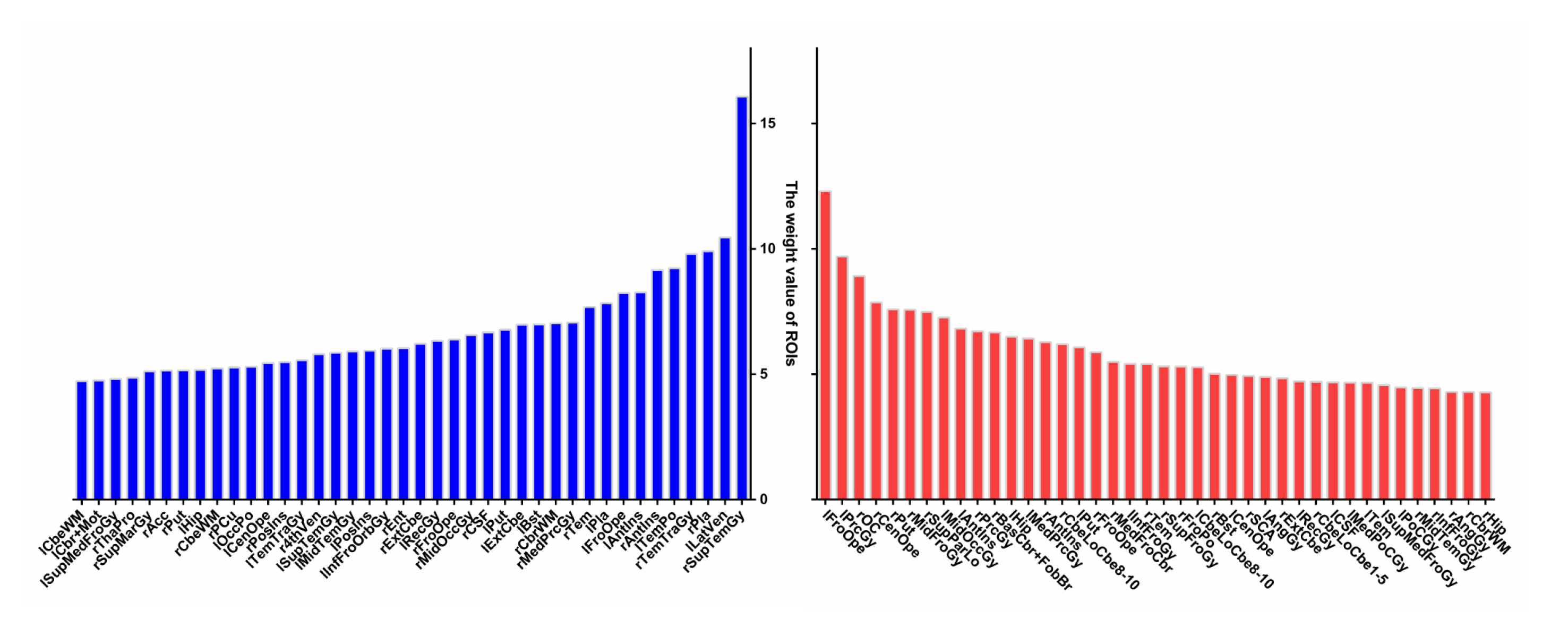


**Supplementary Fig. S14.** Average weight ranking differences of ROI in the subtype-healthy control classification. Subtype1-healthy controls (Left) and subtype2-healthy controls (Right). The average weight of the top 40 ROIs is shown here. Abbreviations: lCbeWM, Left Cerebellum White Matter; lCbr+Mot, Left Cerebrum and Motor; lSupMedFroGy, Left Superior Medial Frontal Gyrus; rThaPro, Right Thalamus Proper; rSupMarGy, Right Supramarginal Gyrus; rAcc, Right Accumbens; rPut, Right Putamen; lHip, Left Hippocampus; rCbeWM, Right Cerebellum White Matter; rPCu, Right Precuneus; lOccPo, Left Occipital Pole; lCenOpe, Left Central Operculum; rPosIns, Right Posterior Insula; lTemTraGy, Left Temporal Transverse Gyrus; r4thVen, Right Fourth Ventricle; lSupTemGy, Left Superior Temporal Gyrus; lMidTemGy, Left Middle Temporal Gyrus; lPosIns, Left Posterior Insula; lInfFroOrbGy, Left Inferior Frontal Orbital Gyrus; rEnt, Right Entorhinal Area; rExtCbe, Right Exterior Cerebellum; lRecGy, Left Gyrus Rectus; rFroOpe, Right Frontal Operculum; rMidOccGy, Right Middle Occipital Gyrus; rCSF, Right CSF; lPut, Left Putamen; lExtCbe, Left Exterior Cerebellum; lBst, Left Brainstem; rCbrWM, Right Cerebral White Matter; rMedPrcGy, Right Medial Precentral Gyrus; rTem, Right Temporal; lPla, Left Planum Polare; lFroOpe, Left Frontal Operculum; lAntIns, Left Anterior Insula; rAntIns, Right Anterior Insula; lTemPo, Left Temporal Pole; rTemTraGy, Right Temporal Transverse Gyrus; rPla, Right Planum Polare; lLatVen, Left Lateral Ventricle; rSupTemGy, Right Superior Temporal Gyrus; lPrcGy, Left Precentral Gyrus; rOC, Right Optic Chiasm; rCenOpe, Right Central Operculum; rMidFroGy, Right Middle Frontal Gyrus; rSupParLo, Right Superior Parietal Lobule; lMidOccGy, Left Middle Occipital Gyrus; rPrcGy, Right Precentral Gyrus; rBasCbr+FobBr, Right Basal Cerebrum and Forebrain Brain; lMedPrcGy, Left Medial Precentral Gyrus; rCbeLoCbe8-10, Right Cerebellar Lobule Cerebellar Vermal Lobules VIII-X; rMedFroCbr, Right Medial Frontal Cerebrum; lInfFroGy, Left Inferior Frontal Gyrus; rSupFroGy, Right Superior Frontal Gyrus; rFroPo, Right Frontal Pole; lCbeLoCbe8-10, Left Cerebellar Lobule Cerebellar Vermal Lobules VIII-X; rBst, Right Brainstem; rSCA, Right Subcallosal Area; lAngGy, Left Angular Gyrus; rCbeLoCbe1-5, Right Cerebellar Lobule Cerebellar Vermal Lobules I-V; lCSF, Left CSF; lMedPoCGy, Left Medial Postcentral Gyrus; lTem, Left Temporal; lPoCGy, Left Postcentral Gyrus; rMidTemGy, Right Middle Temporal Gyrus; rInfFroGy, Right Inferior Frontal Gyrus; rAngGy, Right Angular Gyrus; rHip, Right Hippocampus.

**The quality control of structural images**

***QC of the original T1 image***

(1) At first, an initial quality check has been performed by an experienced radiologist slice by slice. (2) Then the Image Quality Rate (IQR) in CAT12 software has been used in the quality control of structural images to avoid the subjectivity of manual check. IQR was rated by weight average of noise contrast ratio (NCR), inhomogeneity contrast ratio (ICR) and RMS resolution (RES).

***QC of segmentation and normalization***

(1) We manually examined the normalized structural images. (2) We checked the segmented images (gray matter, white matter and CSF) slice by slice to ensure that there were no segmentation errors. (3) The strict IQR exclusion criteria we set in this study ensured the quality of segmentation.

***QC of the ROI volumes***

(1) We checked the alignment between the Neuromorphometrics atlas and normalized volumetric maps slice by slice by overlaying the Neuromorphometrics atlas on the normalized volumetric maps.

(2) We checked the segmented ROI volumes to see if there were any absurd values (0 or NaN).

(3) Through the self-contained outlier detection function of HYDRA, the outlier can be observed through the subtyping process. In other words, the number of clusters ranges from 2 to 8. If some subjects had abnormal ROI volumes values, that is, there were outliers, we would observe a rising in ARI value when the number of clusters K increased. In this paper, ARI did not increase as K increased, which indicated that there were no outliers.

**Image quality measures in CAT12**

The image quality measures were estimated by using the tissue segmentation and were (nearly) independent of subject sex and health status. There are three image quality measures:

(1) *NCR:* the NCR measures the local standard deviation in the optimized WM segment and is scaled by the minimum tissue contrast.

(2) *ICR:* the ICR is measured as the global standard deviation within the optimized WM segment and is scaled by the minimum tissue contrast.

(3) *RMS:* to describe the resolution of an image, the RMS value of the voxel size is used. Outliers with exceptionally low resolutions in one of the x, y or z dimensions are weighted much stronger than outliers with high resolutions, resulting in an asymmetric penalization.

The final resulting ratings, image quality rating (IQR) were combined as weighted average by the above three measures.

**Resting-state fMRI data processing**

The resting-state image preprocessing was carried out using Data Processing Assistant for Resting-State fMRI (DPARSF, http://rfmri.org/DPARSF), which is based on Statistical Parametric Mapping (SPM12, <http://www.fil.ion.ucl.ac.uk/spm/>). The main steps applied to the fMRI data were as follows: (1) removal of the first 10 time points; (2) slice timing correction; (3) realignment; (4) structural MRI data were co-registered to fMRI data; (5) normalization to the MNI coordinate space with voxel size resampled into 3 × 3 × 3 mm^3^; (6) detrend; (7) regression of 24 head motion parameters, white matter, and CSF signals; (8) bandpass filtering (0.01-0.1 Hz);

**Brief introduction of R-fMRI measures**

1) Regional homogeneity (ReHo) ^1^. ReHo assesses the degree of regional synchronization/coherence among fMRI time courses. It is defined as the Kendall’s coefficient of concordance (KCC) between the time series of a given voxel and its nearest neighbors (In the current study ReHo was calculated as KCC of a given voxel with its 26 adjacent voxels). 2) Homotopic interhemispheric connectivity (VMHC) ^2,3^. VMHC is defined as the Pearson correlation coefficient of BOLD signal time series of specific voxels and voxels at the same position in the contralateral hemisphere. VMHC requires that individual functional data are first registered in MNI space and smoothed (FWHM = 4 mm) and registered to a symmetric template ^2^. Then, the VMHC values were Fisher-Z transformed. 3) Degree centrality (DC) ^4,5^. DC is the number or sum of weights of significant connections for a voxel. Here, we calculate the functional connection (FC) of voxel level and whole brain voxel level one by one, and then take the positive connection exceeding the threshold (In this study threshold was set as Pearson correlation coefficient r > 0.25) in FC to calculate the weighted sum to obtain the whole brain DC mapping of voxel level ^4^. 4) Global Signal correlation (GSCorr): GSCorr first calculates the average time series of all voxels in the group mask as the global signal, and then calculates the Pearson correlation coefficient between the voxel time series and the global signal voxel by voxel in the group mask. These correlation values were then Fisher-Z transformed.

**Dynamic** **R-fMRI measures** **calculation**

In order to compare whether there are differences in the dynamic characteristics of brain spontaneous activity among different subtypes of ASD, 3 measures were calculated by sliding time window method based on Temporal Dynamic Analysis in Data Processing & Analysis for Brain Imaging (DPABI, http://rfmri.org/DPABI). First, we applied hamming windows (length of 45 TRs, sliding step of 4 TRs) to BOLD signals to obtain windowed time series; Then, within each window, we calculated the above-mentioned R-fMRI indices (i.e., ReHo, VMHC, DC and GSCorr). To quantitatively characterize the dynamic R-fMRI measures, we compute the standard deviation (SD) map across time windows for each measure. The SD maps were then Z-standardized by its own mean and SD across all the whole brain voxels within the group mask. Finally, we smoothed (FWHM = 4 mm) the SD map of each measure (Except VMHC, it has been smoothed in processing) to improve the signal-to-noise ratio.

**Dynamic functional stability measure** **calculation**

For a given voxel, the within-state stability of dynamic functional architecture was defined as the concordance of dynamic functional connection (DFC) over time of that voxel with the whole brain. DFC was calculated over consecutive segments of data using a sliding-window approach ^6^ based on Stability Analysis in DPABI, the window length and the sliding step are consistent with the calculation of the above dynamic R-fMRI measures. We conducted analyses in a voxel-to-atlas approach, such that DFC was computed between a voxel with 142 parcellations from the Neuromorphometrics atlas. Functional stability of a voxel in the brain was calculated as KCC of DFC between that voxel with all parcellations.

**References**

1. Zang Y, Jiang T, Lu Y, He Y, Tian L. Regional homogeneity approach to fMRI data analysis. *Neuroimage.* 2004;22(1):394-400.

2. Zuo XN, Kelly C, Di Martino A, et al. Growing together and growing apart: regional and sex differences in the lifespan developmental trajectories of functional homotopy. *J Neurosci.* 2010;30(45):15034-15043.

3. Anderson JS, Druzgal TJ, Froehlich A, et al. Decreased interhemispheric functional connectivity in autism. *Cereb Cortex.* 2011;21(5):1134-1146.

4. Buckner RL, Sepulcre J, Talukdar T, et al. Cortical hubs revealed by intrinsic functional connectivity: mapping, assessment of stability, and relation to Alzheimer's disease. *J Neurosci.* 2009;29(6):1860-1873.

5. Zuo XN, Ehmke R, Mennes M, et al. Network centrality in the human functional connectome. *Cereb Cortex.* 2012;22(8):1862-1875.

6. Hutchison RM, Womelsdorf T, Allen EA, et al. Dynamic functional connectivity: promise, issues, and interpretations. *Neuroimage.* 2013;80:360-378.
